# Identification of key lncRNAs contributing to diabetic nephropathy by gene co-expression network analysis

**DOI:** 10.1038/s41598-019-39298-9

**Published:** 2019-03-01

**Authors:** Jin Shang, Shuai Wang, Yumin Jiang, Yiqi Duan, Genyang Cheng, Dong Liu, Jing Xiao, Zhanzheng Zhao

**Affiliations:** 1 Department of Nephrology, the First Affiliated Hospital of Zhengzhou University, Zhengzhou, 450052 P.R. China; 2 Department of Emergency, the First Affiliated Hospital of Zhengzhou University, Zhengzhou, 450052 P.R. China; 3 Department of Pharmacy, the First Affiliated Hospital of Zhengzhou University, Zhengzhou, 450052 P.R. China

## Abstract

LncRNA is reported to have important role in diabetic nephropathy (DN). Here, we aim to identify key lncRNAs of DN using bioinformatics and systems biological methods. Method: Five microarray data sets from Gene Expression Omnibus (GEO) database were included. Probe sets were re-annotated. In the training set, differential expressed genes (DEGs) were identified. Weighted gene co-expression network analysis (WGCNA) was constructed to screen diabetic-related hub genes and reveal their potential biological function. Two more human data sets and mouse data sets were used as validation sets. Results: A total of 424 DEGs, including 10 lncRNAs, were filtered in the training data set. WGCNA and enrichment analysis of hub genes showed that inflammation and metabolic disorders are prominent in DN. Three key lncRNAs (NR_130134.1, NR_029395.1 and NR_038335.1) were identified. These lncRNAs are also differently expressed in another two human data sets. Functional enrichment of the mouse data sets showed consistent changes with that in human, indicating similar changes in gene expression pattern of DN and confirmed confidence of our analysis. Human podocytes and mesangial cells were culture *in vitro*. QPCR and fluorescence *in situ* hybridization were taken out to validate the expression and relationship of key lncRNAs and their related mRNAs. Results were also consistent with our analysis. Conclusions: Inflammation and metabolic disorders are prominent in DN. We identify three lncRNAs that are involved in these processes possibly by interacting with co-expressed mRNAs.

## Introduction

Chronic kidney disease (CKD) has become a worldwide public health problem which is associated with high mortality and heavy economic burden^1^. As the incidence of diabetes increasing, diabetic nephropathy (DN), the major microvascular complication of diabetes mellitus, is becoming the major cause of end-stage renal disease especially in developed countries^2^. Certain advances about the molecular mechanism of DN have been made during last decades. However, our understanding about the disease is still limited and the treatment is mainly based on control of blood pressure and glucose, and blocking of the renin-angiotensin system^3^. Previous studies were mainly based on technology of molecular biology which only focused on limited number of molecules. During DN, lots of genes will change in a coordinate way. As a result, genomic approaches are needed to describe the changes in gene expression profiles.

Long noncoding RNA (lncRNA) is a class of transcripts that are longer than 200 nucleotides and cannot coding proteins. The mammalian genomes have plenty of lncRNAs which are engaged in different biological processes by participating DNA methylation, mRNA transcription, alternative splicing, maturation, decay and translation^4,5^. LncRNAs are often poorly conserved among different species. Previous studies showed some important lncRNAs may be involved in DN, including metastasis-associated lung adenocarcinoma transcript 1 (MALAT1), plasmacytoma variant translocation 1 (PVT1) and taurine up-regulated gene 1 (TUG1)^6–8^. However, none of the studies analyzed those using methods of bioinformatics and systems biology.

The goal of this study was to identify key lncRNAs and their roles in DN from the aspect of whole gene transcripts analysis. We used data downloaded from Gene Expression Omnibus (GEO) database. A human microarray data set was used as training set. Differential expressed genes (DEGs) were screened. Weighted gene co-expression network analysis (WGCNA), a widely used systems biological method for finding gene sets that are highly correlated with clinical traits, was constructed to screen gene modules that were highly co-regulated and closely related with DN within in modules. Then gene co-expression analysis was used to find hub lncRNAs and reveal their potential biological function by showing lncRNA-mRNA interactions. Two more human data sets and mouse data sets were used as validation sets. We also validated the expression and relationship of the screened lncRNAs and mRNAs using qPCR and Fluorescence *in situ* hybridization (FISH). To the best of our knowledge, it is the first time of analyzing combined DN data sets to locate key lncRNAs using bioinformatics and systems biological methods.

## Results

### Multiple data sets were included

Finally, 5 series (GSE30122, GSE47183, GSE47184, GSE20636 and GSE33744, Table 1) were screened out according to our filter criteria^9–14^. Three of them (GSE30122, GSE47183 and GSE47184) used human samples while the other two of them (GSE20636 and GSE33744) used mouse samples. GSE30122 was used as a training set which contains 10 diabetic tubuli (TD), 24 control tubuli (TC), 9 diabetic glomeruli (GD) and 26 control glomeruli (GC). The other four were used as validation data set.

**Table 1:** Summary of included data sets.

Series	Platform	Sample size	Diabetic Nephropathy		Tissue			Species
			Diabetic	Control	Glomeruli	Tubuli	Whole Kidney	
GSE30122	GPL571	69	19	50	35	34	—	Homo sapiens
GSE47183	GPL11670, GPL14663	122	14	—	14	—	—	Homo sapiens
GSE47184	GPL11670, GPL14663	107	18	4	—	22	—	Homo sapiens
GSE20636	GPL1261	35	19	16	—	—	35	Mus musculus
GSE33744	GPL1261	39	21	18	39	—	—	Mus musculus

### Differential expressed mRNAs and lncRNAs were screened out

**Figure 1:**
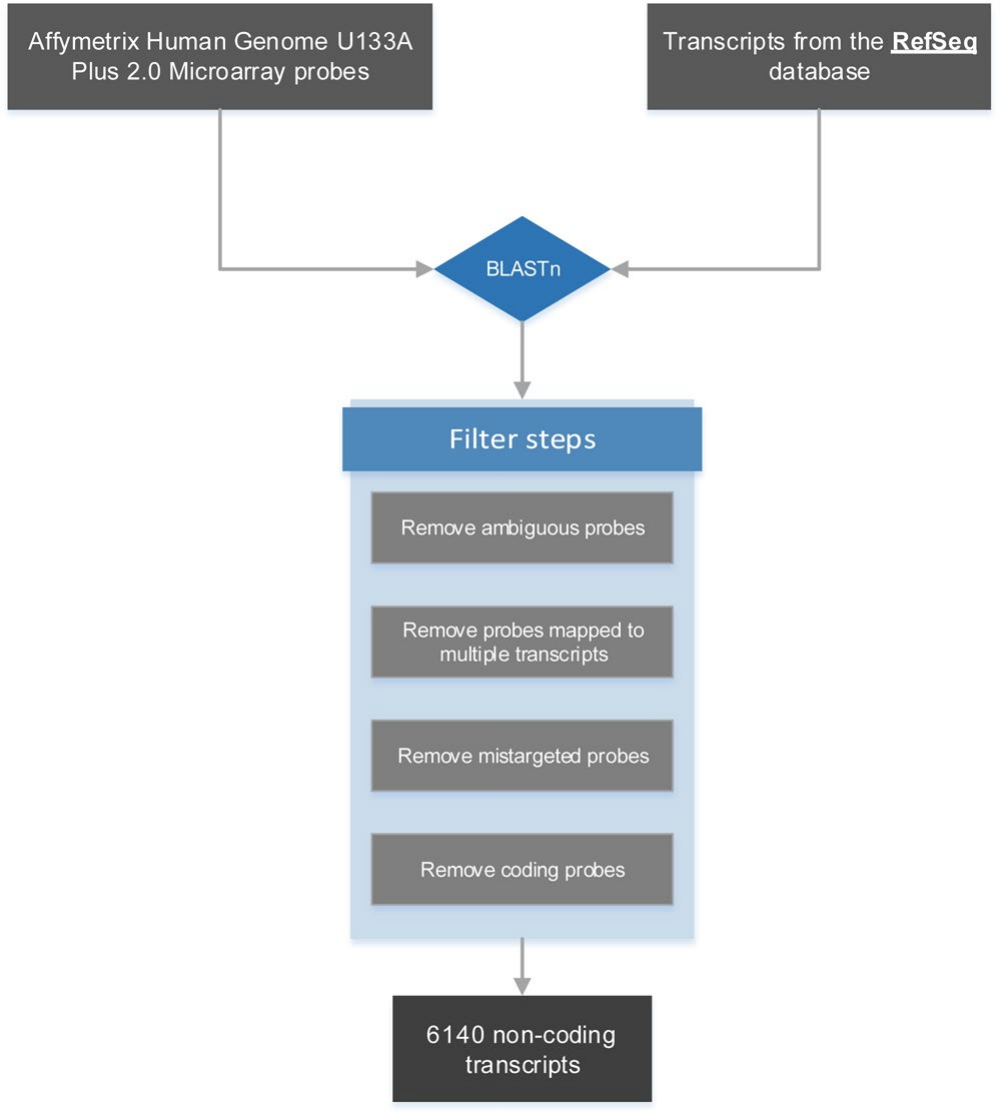
LncRNA reannotation pipeline.

**Figure 2:**
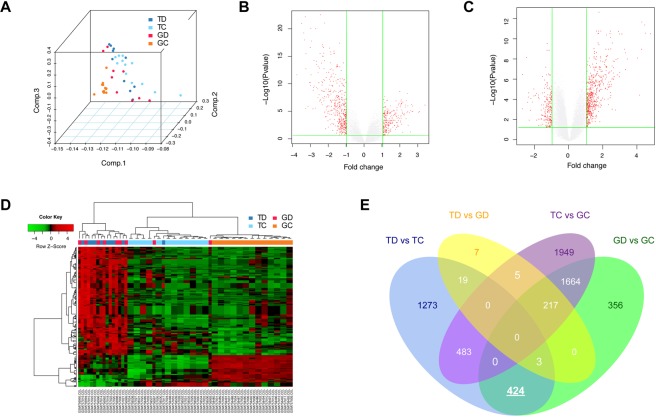
Characterizations of expression profiles within different groups. The training dataset (GSE30122) were downloaded from GEO database (https://www.ncbi.nlm.nih.gov/geo/). Principle component analysis of samples in 4 groups was shown in (**A**). (**B**) Volcano plot of differential expressed genes (DEGs) between diabetic tubuli (TD) and control tubuli (TC) samples. x axis corresponds to log2 transformed fold change, and y axis corresponds to –log10 transformed P value. (**C**) Volcano plot of DEGs between diabetic glomeruli (GD) and control glomeruli (GC) samples. (**D**) Heatmap of DEGs among 4 groups. (**E**) Venn plot of DEGs among 4 groups.

A modified lncRNA reannotation pipeline was used to re-annotate probe sets in each data set (Fig. 1). Characterizations of expression profiles within different groups of the training data set were shown in Fig. 2. Principle component analysis gives us an overview of the internal structure of sample data in four groups. DEGs between TD and TC, GD and GC were shown in volcano plots. DEGs among 4 groups were illustrated and clustered in heatmap. After intersecting DEGs in both TD and GD compared with TC and GC, and removing potential irrelevant genes, 424 genes, including 10 lncRNAs, were finally filtered and selected for subsequent analysis. Venn plot was shown in Fig. 2E. DELs and DEGs were shown in Supplementary Tables S1 and S2.

### WGCNA was used to find out diabetic-associated gene modules

**Figure 3:**
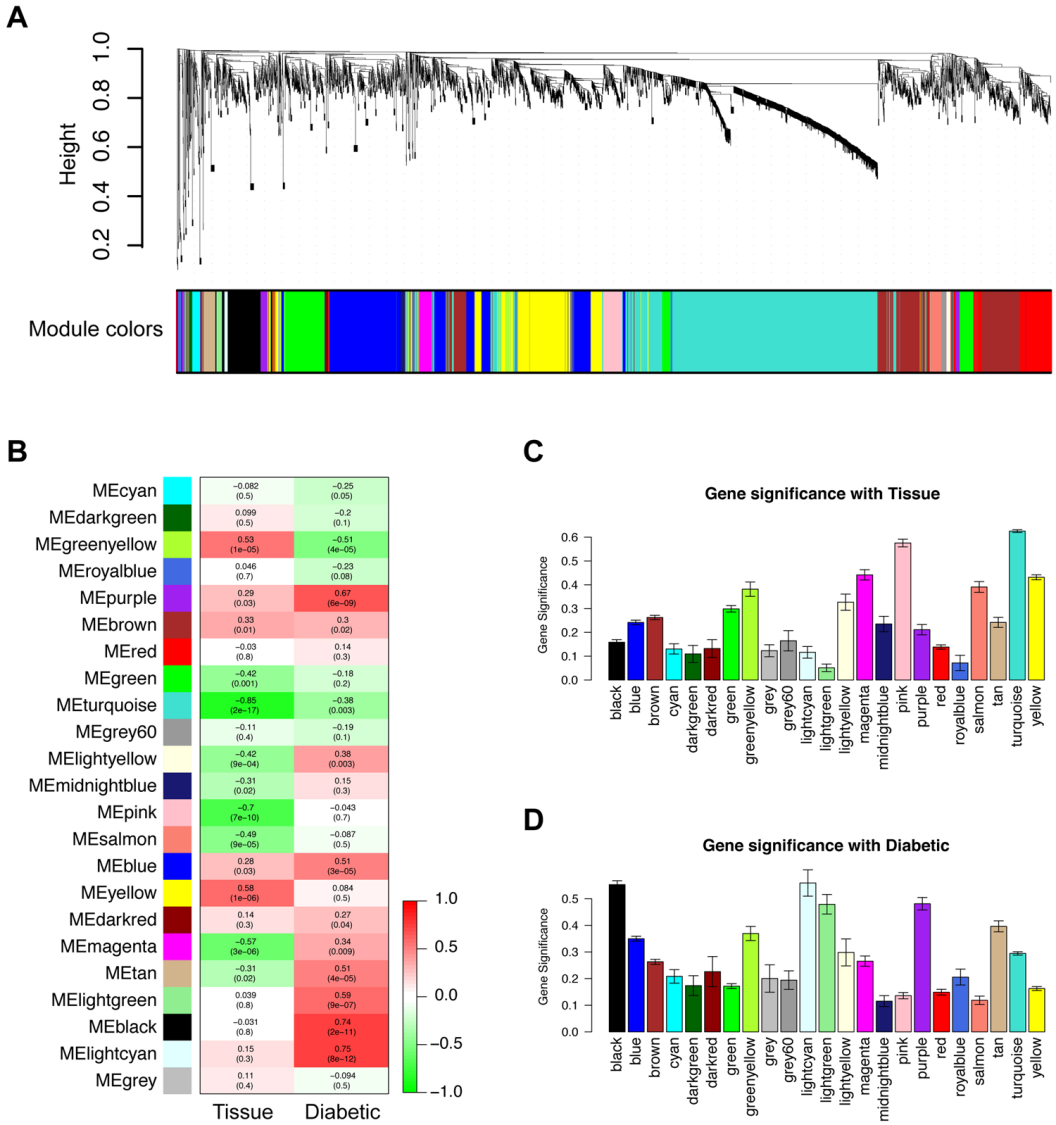
Genes modules in weighted gene co-expression network analysis (WGCNA) analysis. (**A**) Module assignment in hierarchical clustered genes. Genes within different modules are labeled with different colors according to WGCNA’s conventions. (**B**) Heatmap of correlations between modules and clinical traits, including tissue type (glomeruli or tubuli) and phenotype (diabetic or not). Colors corresponds to correlations, while red means positively correlated and green means negatively correlated. Correlations values and P values are also labeled. Tissue type (**C**) and diabetic (**D**) associated gene significance of different modules. The top three gene modules most significantly related with diabetic (black, lightcyan, purple) were selected for further analysis. Y axis represents the significance values.

**Figure 4:**
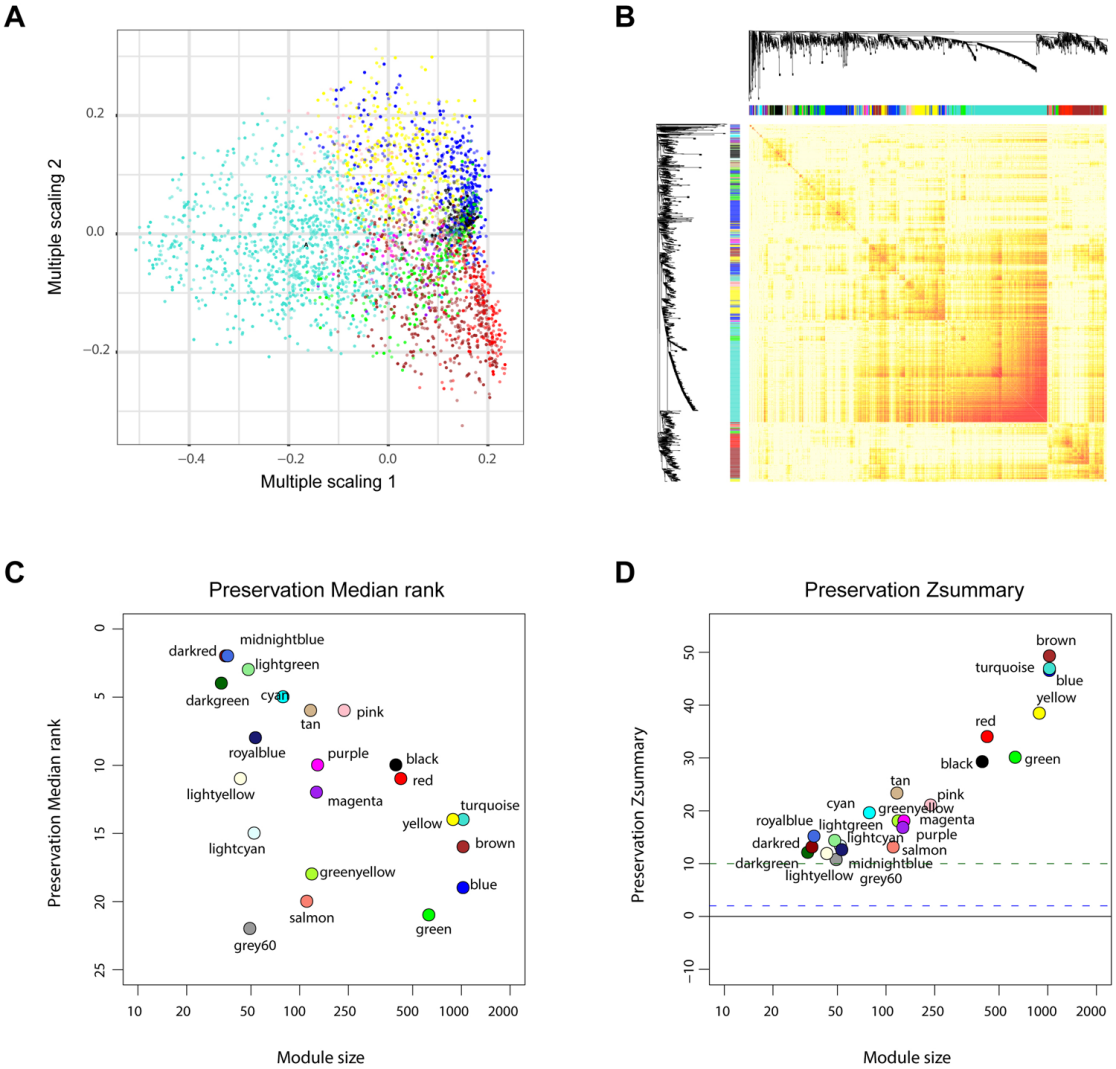
Characterizations of gene modules in WGCNA. (**A**) Multiple scaling plot showing gene clusters. (**B**) Topological overlap matrix (TOM) plot showing pairwise gene correlations within each module. Module preservations median rank (**C**) and Zsummary score (**D**) of all modules were presented. The median Rank of the modules close to zero indicates a high degree of module preservation. The dashed blue and green lines indicate the thresholds Z = 2 and Z = 10 respectively.

Samples were first clustered to remove outlined samples. (A dendrogram was shown in Supplementary Fig. S1). WGCNA was then performed to identify genes that were highly co-expressed across groups. Modules were randomly color-labeled. A total of 24 gene modules were identified (Fig. 3A). Then module eigengene were analyzed for correlation with tissue or disease traits. Module eigengene-to-condition correlation heatmap were shown in Fig. 3B. Since the data set includes samples from both glomeruli and tubuli, we analyzed which gene modules were clustered due to tissue differences. Figure 3C illustrate the gene significance of each module with tissue and the most significant ones were pink (r = −0.7, p = 7e-10) and turquoise (r = −0.85, p = 2e-17). As for diabetic phenotype, the top three correlated modules are lightcyan (r = 0.75, p = 8e-12), black (r = 0.74, p = 2e-11) and purple (r = 0.67, p = 6e-09) (Fig. 3D). Interestingly, their correlations with tissue are relatively weak, indicating important roles in DN process. As a result, they were selected as module of interest and subjected to following analysis. To verify the robustness of WGCNA, we analyzed the module conservation. The multiple scaling plot (Fig. 4A) shows gene clusters labeled by module colors. Topological overlap matrix (TOM) plot were constructed to show the pairwise gene correlation within each module (Fig. 4B). The stability of modules was calculated. Preservation median rank and preservation Z summary score of all modules were presented in Fig. 4C,D. The three diabetic-related modules are relatively stable since their median rank preservation was relatively low and the Z summary statistics were above the threshold of 10. Exact value could be found in Supplementary Table S3.

### Hub genes and CNC network construction reveal three key lncRNAs

**Figure 5:**
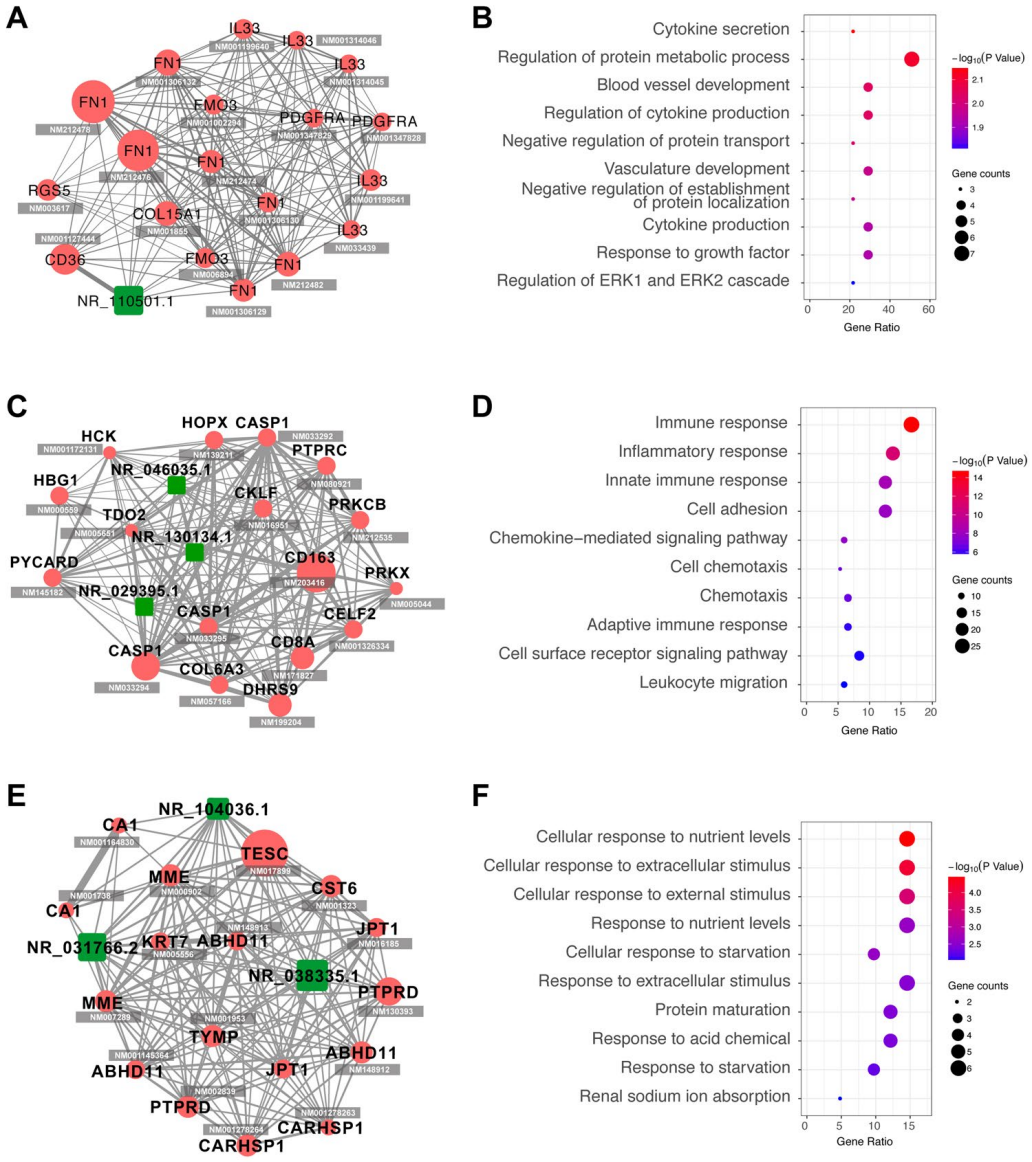
Hub genes and their functions in diabetic-correlated gene modules. Gene modules most significantly correlated with diabetic (as shown by WGCNA results) were selected for further analysis. Network of hub genes in lightcyan (**A**), black (**C**) and purple (**E**) modules were constructed. Red node represents protein coding gene and green node represents lncRNA. Line width corresponds to gene correlation and node size corresponds to its hub score. RefSeq Accession number is listed below each gene symbols. Molecular function of genes in lightcyan (**B**), black (**D**) and purple (**F**) modules were enriched using the online tool DAVID. Color of circle corresponds to −log10 transformed P values, and circle size corresponds to count of genes in the GO term.

In selected modules, genes were ranked using formula described in method part and the top 20 were considered as hub genes. CNC network and GO enrichment analysis of hub genes were constructed. Figure 5A,C,E showed that each network contains at least one lncRNA which forms intensive connections with other mRNAs. In lightcyan module (A, B), NR_110501.1 is the key lncRNA. Regulation of protein metabolic process is prominently enriched. In black module (C,D), lncRNA NR_046035.1, NR_130134.1 and NR_029395.1 are involved. DEGs are mostly enriched in immune-related processes, including immune response, inflammatory response and innate immune response. The purple module (E, F) involves lncRNA NR_104036.1, NR_031766.2 and NR_038335.1. Metabolism (cellular response to nutrient levels, response to nutrient levels, cellular response to starvation, protein maturation and response to starvation) and stress (cellular response to extracellular stimulus, cellular response to external stimulus, response to extracellular stimulus and response to acid chemical) -related pathways are intensively enriched. Different functional enrichment indicates that these lncRNAs participated in different biological processes by interacting with various mRNAs. As we noticed that three of the lncRNAs (NR_130134.1, NR_029395.1 and NR_038335.1) were also the DELs shown in Supplementary Table S1, indicating their important roles in DN. Detailed information about the functional enrichment could be found in Supplementary Table S4.

### DELs validation in human data sets

**Figure 6:**
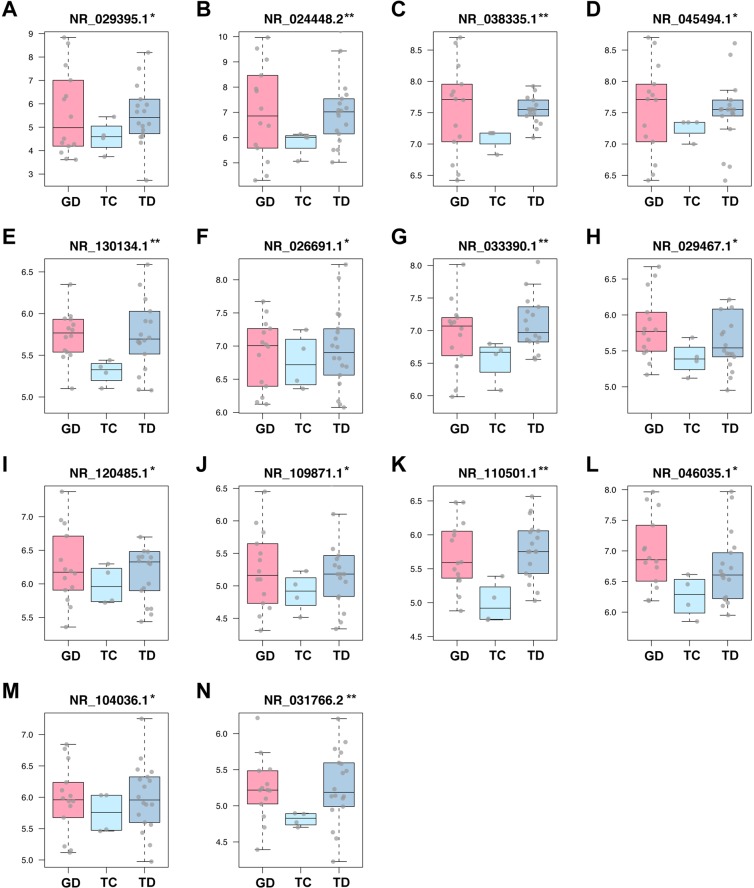
Expression values of differential expressed lncRNAs (DELs) in different groups of GSE47183 and GSE47184. The other two data sets of homo sapiens (GSE47183 and GSE47184) downloaded from GEO were analyzed to validate the DELs of the training set. A total of 36 samples containing GD, TC and TD were included. After batch effects removal, DELs with statistical significant difference were shown and masked using asterisk. X axis was labeled with tissue type, while y axis was log2 transformed expression level. (*P < 0.05, **P < 0.01).

To further validate the three key lncRNAs screened from the training data set, we compared them with the DELs in the validation data sets (GSE47183 and GSE47184). Batch effects of expression values were removed (see Supplementary Fig. S2). Box plots of DELs were shown in Fig. 6, among which, the three key lncRNAs of the training data set showed up again.

### Changes of gene expression pattern are similar in human and mouse data sets

**Figure 7:**
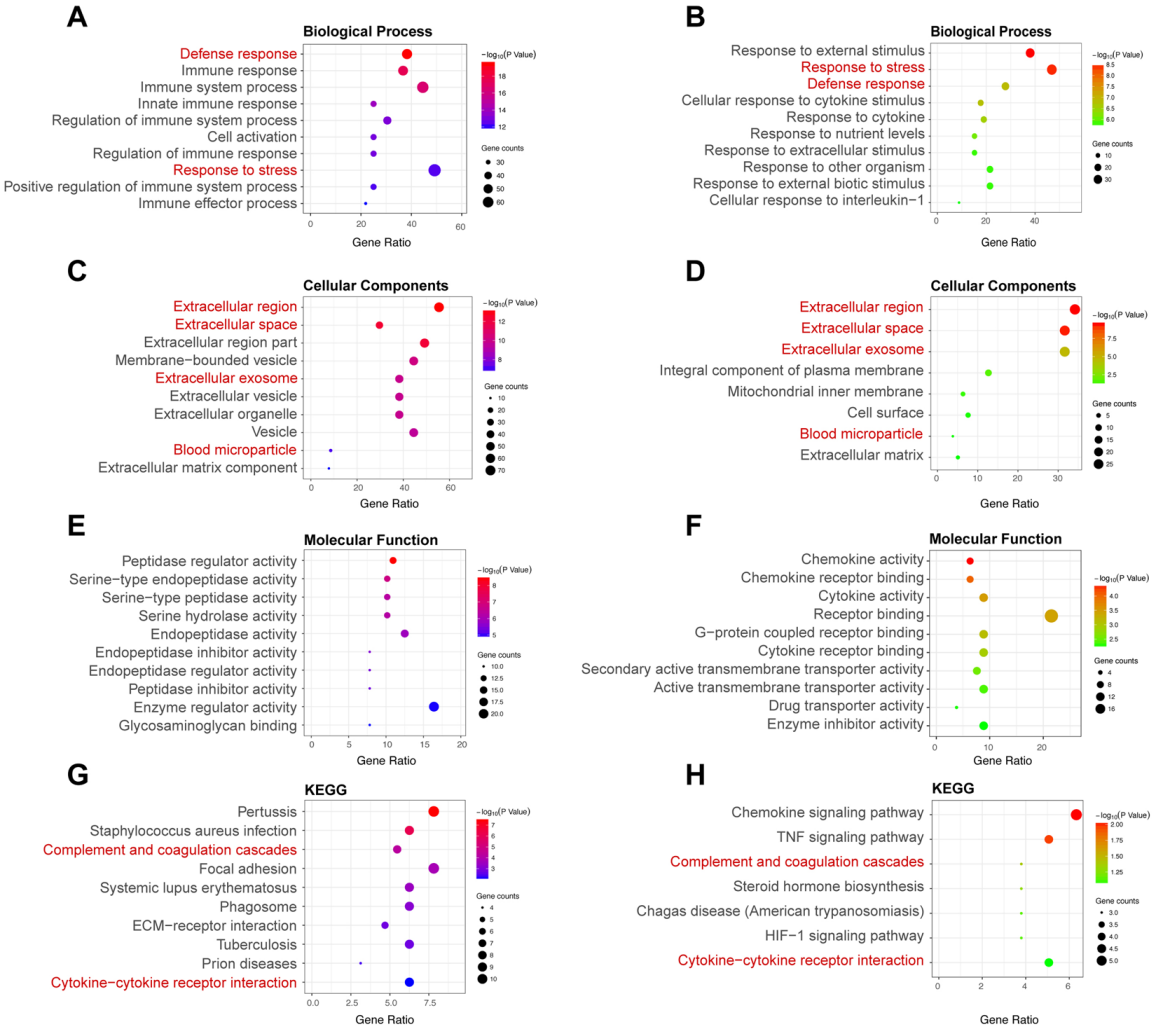
Compare of function enrichment of DEGs between DN and control in human and mouse. Gene ontology, including biological process (**A**), Cellular components (**C**), Molecular function (**E**), and KEGG pathway (**G**) enrichment analysis of DEGs among diabetic and control samples in human. Gene ontology, including biological process (**B**), Cellular components (**D**), Molecular function (**F**), and KEGG pathway (**H**) enrichment analysis of DEGs among diabetic and control samples in mouse. Color of circle corresponds to –log10 transformed P values, and circle size corresponds to count of genes in the GO term.

LncRNAs are poorly conserved in different species. It is difficult to compare lncRNA directly between different species. As a result, we compared DEGs and the gene function changes between human data sets and two mouse data sets (GSE20636 and GSE33744). Differential expression analysis was carried out and 172 recurrent DEGs in mouse data sets were filtered (see Supplementary Table S5). To further explore the similarity of DEGs identified in human and mouse, functional enrichment analysis was also performed. Significant enriched terms or pathways were illustrated in Fig. 7 and also listed in Supplementary Tables S6 and S7. Identical pathways between species were represented in red which indicated similar changes in gene expression pattern of DN and showed confidence of our analysis. A,C,E and G are results of human and B,D,F and H are results of mouse.

### Validation of the DELs and related mRNAs

**Figure 8:**
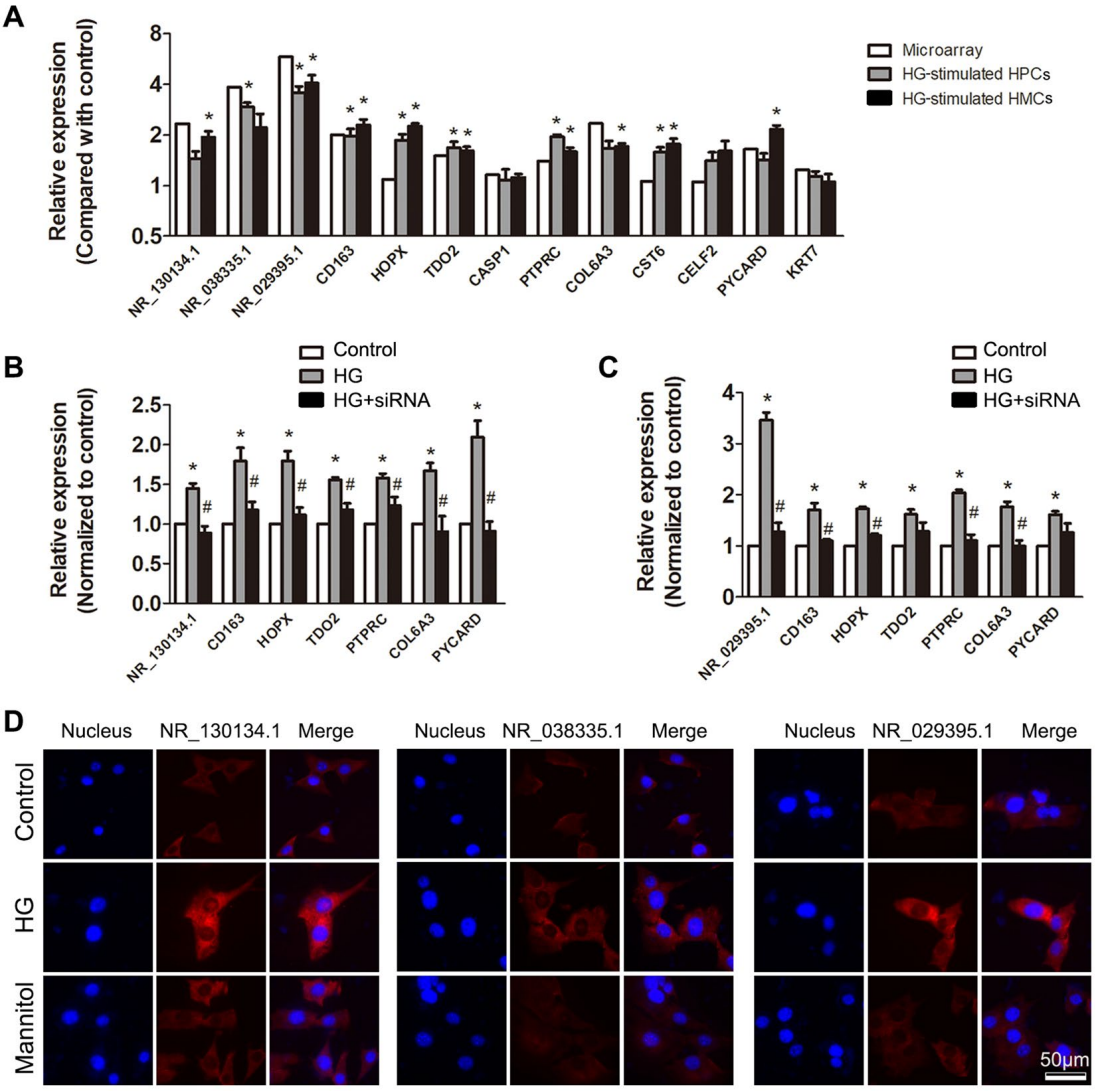
Validation of expression and relationship of the key genes. (**A**) HPCs and HMCs were cultured *in vitro* and stimulated by high glucose. Expression of the key lncRNAs and some of their co-expressed mRNAs were tested by qPCR. Relative expression in high glucose group was normalized to expression in normally cultured cells. LncRNA NR_130134.1 (**B**) and NR_029395.1 (**C**) were knocked down by specific siRNA in cultured HMCs. After that, high glucose (40 mM) was added for 24 h. Expression of related mRNAs was tested in each group. n = 3, *P < 0.05 vs. Control group. ^#^P < 0.05 vs. high glucose group. (**D**) Expression of three key lncRNAs was tested by FISH in high glucose-stimulated HMCs.

The three key lncRNAs were involved in two networks as shown in Fig. 5. Related genes that were differentially expressed in DN glomerulus were chosen for further validation. We cultured HPCs (human podocytes) and HMCs (human mesangial cells) *in vitro* and stimulated them with high glucose. Results of qPCR showed that expression of three lncRNAs and their co-expressed genes was similar with results of microarray (Fig. 8A). Next, we transfected siRNAs targeting lncRNA NR_130134.1 and NR_029395.1 into HMCs to further validate their relationship with other DEGs. Generally suppressed expression of most mRNAs in siRNA group suggested the central roles of lncRNAs (Fig. 8B,C). FISH was used to show the expression and localization of three lncRNAs in high glucose-stimulated HMCs (Fig. 8D).

## Discussion

At present, high-throughput microarray technology has been used to identify changes in gene expression profiles associated with the onset and progression of DN^15,16^. During DN, functionally related genes have similar changes in their expression patterns^17^. However, most studies focused on individual differential expressed genes and ignored the changes in gene interconnection networks. Genome wide transcript analysis can descript changes in gene expression pattern and now has been widely used in disease studies^18,19^.

LncRNA is a class of molecules that have been reported to participate in the onset and progression of DN^6,20,21^. Previous studies mainly showed their functions using molecular biologic method. LncRNAs could form complex networks with plenty of genes. Understanding the molecular interactions among key lncRNAs and mRNAs are essential for diagnosis and treatment of DN. As a result, it is necessary to use systems biology method in lncRNA screening and function interpretation of genome-wide expression data sets. However, multiple issues, including different disease staging, system error due to different research batches, different species used in different studies and lack of mouse models that would faithfully simulate changes of DN will lead to different conclusions^22^. Therefore, a comprehensive analysis of data containing multiple data sets from different species would be necessary.

In this study, we included five GEO data sets. Each of them contains samples more than 30 and derived from human or mice kidney tissue. Among them, gene expression profiling of GSE30122 that includes both tubular and glomerular samples of control or DN patients was used as training dataset. PCA showed that different groups clustered differently, indicating changes in gene expression profiling. As we expected, large number of DEGs were found, including 10 DELs.

WGCNA is a systems biology method used for finding modules of highly correlated genes and identifying phenotype related gene clusters. It is widely used in identifying the correlations between gene sets and clinical characteristics^23^. By constructing a gene co-expression network, we can find intra-modular hub genes and provide global information about hub gene-associated expression profiles as well as gene functional relationship. Here we use WGCNA to identify gene modules that showed highly correlation patterns with diabetic nephropathy across four groups (DG, TG, GC, TC). A total of 24 modules were identified. To find out modules that best correlated with diabetic state, the module-to-condition correlations were constructed and visualized as heatmap. Glomeruli and tubuli exhibit different expression profiles due to different cellular inhabitants. As a result, it is not surprising that some modules are associated with tissue difference. However, when tissue-caused variations are small, several modules are still significantly correlated with diabetic phenotype. As we know, glomerular damage is significant in diabetic nephropathy. Our results show that some gene modules change in both glomeruli and tubuli, suggesting diabetic tubuli damage should be focused in future study. The top three diabetic-associated modules are colored by lightcyan, purple and black.

Next, we analyzed the network connectivity and module conservation. TOM analysis was used which could give us a reduced view of the whole network. The connectivity of a gene is the sum of its adjacency with other genes. Nodes that are highly interconnected show high topological overlap and are represented by a darker color shade. The rows and columns are ordered by the WGCNA color bar which is shown on the left and top of the TOM plot. Preservation median rank and preservation Z summary score of all modules were presented and results suggested those three modules showed acceptable conservation. As a result, they are selected as module of interest for subsequent analyses.

Genes within a module are closely related in function. The lncRNAs involved is therefore supposed to have the similar functions. To screen the key lncRNAs, we constructed expression network and functional enrichment. In the three modules that are significantly associated with diabetic trait, a total of seven hub lncRNAs were found. From the enrichment analysis, we could see that these lncRNAs in each module participated in different biological processes. DN is known to be a metabolic problem such as energy, protein and lipid disorder^24,25^. Clinical trials have suggested that nutritional and dietary interventions in DN are an essential aspect of management and patients will benefit from a strict dietary protein and carbohydrates control^26^. Our results of function enrichment of modules also indicated an important role of nutrition and metabolic process. LncRNAs (especially NR_110501.1, NR_104036.1, NR_031766.2 and NR_038335.1) seem to be a participator of these processes. Besides metabolism problems, immune and inflammatory disorders are also believed to be involved^27^. Complement and immunoglobulins sometimes can also be detected in diseased glomeruli of DN^28^. However, due to serious side effects, usage of immunosuppressive drugs is limited in DN therapy. Recently, studies indicated that lncRNA might be a promising novel target for managing DN inflammation^20^. Here we show that, besides mRNAs, several lncRNAs (NR_046035.1, NR_130134.1 and NR_029395.1) are involved in immune and inflammatory responses of DN, which might hopefully provide us new targets for DN treatment. By comparing the hub genes with the DELs, we found three identical genes: NR_130134.1, NR_029395.1 and NR_038335.1, which might be the key lncRNAs in DN onset.

Then we searched for the DELs in other two human data sets which contain three groups: GD, TC and TD. A total of 14 lncRNAs were screened out. After removing the batch effect, the differences still existed (see Supplementary Fig. S2). What’s worth noticing is that the three key lncRNAs also shows up here, suggesting the robustness of these differences among data sets.

To testify the accuracy of our results, we analyzed two data sets of mice. A total of 172 recurrent DEGs showed up. However, no consistent lncRNAs was reappeared. The reason might involve that lncRNAs are poorly conserved and their sequences among different species are very different. Then we compared the functional enrichment of DEGs. Results showed some identical pathways which were highlighted in red font. These validated the robustness of gene changes found in human data sets and proved the mechanism of DN might be similar among species. However, major differences in gene functions still exist. As a result, when we interpret mechanism of DN using mouse models, we should consider species differences.

HPCs and HMCs are two main kinds of parenchymal cells that have important function in DN. As a result, we cultured them for experimental validation. Results of qPCR and FISH showed that most of the lncRNAs and mRNAs had similar expression pattern with that of microarray, although some might express differently in different cells. Gene interference results also suggested that lncRNAs had close relationship with the expression of related mRNAs. These results indicated that lncRNAs may regulate expression of mRNAs in a direct or indirect way which worth further study.There are some limitations in this study. The data sets included are all microarray data with relatively small sample size. With the development of RNA sequencing technology, the results need to be validated. In another way, lncRNA was defined by reannotation and comparing with lncRNA database. This may lead to some inaccuracy which could not be avoided at present. As a result, more studies would be needed in the future.

In summary, the key finding of this study is that we identified diabetic-related gene expression profiles using bioinformatics method. Inflammation and metabolic disorders seem to be much more prominent biological changes in DN. Three key lncRNAs was found and validated using different methods and in different data sets. They may play important roles in DN by regulating inflammation and metabolism. This may help us understanding the mechanism of DN and provide us new biomarkers for diagnosis, risk stratification and prognosis prediction in DN patients.

## Materials and Methods

### Material and data

We used “diabetic nephropathy” and “diabetic kidney disease” as key words to retrieve data sets from the National Center of Biotechnology Information (NCBI) GEO database (https://www.ncbi.nlm.nih.gov/geo/), and found 59 potential data series. Data series were reviewed one after another using exclusion criteria below: (1) Sample size larger than 30; (2) Expression profiles derived from kidney tissue, not serum or cell lines; (3) Uniform platform used for quantification; (4) Organisms should be Homo sapiens or Mus musculus.

### LncRNA reannotation

A modified lncRNA reannotation pipeline (Fig. 1) was used to re-annotate probe sets in each data set. In summary, four different microarray platforms were used, which were Affymetrix Human Genome U133 Plus 2.0 Array (HGU133Plus2), Affymetrix Human Genome U133A array (HGU133A), Affymetrix Human Geonme U133A 2.0 Array (HGU133A_2) and Affymetrix Mouse Genome 430 2.0 Array (MG430_2). After reannotation, 6160 lncRNAs in HGU133A, 6140 lncRNAs in HGU133A_2, 15268 lncRNAs in HGU133Plus2, and 3882 lncRNAs in MG430_2 were identified.

### Differential expression analysis

Raw probe intensities were normalized and background adjusted by using LIMMA R package^29^. According to the sample information, batch effectors were identified and removed using R sva package^30^. DEGs were identified based on fold changes and P values. A fold change threshold of ≥2.0 and P value ≤ 0.05 were set for up- and down regulated genes. Differential expression analysis was performed between all comparative conditions. Functional enrichment analyses including Gene Ontology (GO) and Kyoto Encyclopedia of Genes and Genomes (KEGG) pathway enrichment were assessed using the DAVID database (https://david.abcc.ncifcrf.gov/)^31^. For functional enrichment analysis of DEGs, the background was set to the total list of genes expressed in humans. The statistically significant threshold level for enrichment analyses was P ≤ 0.05 (Benjamini and Hochberg corrected for multiple comparisons). Hierarchical clustering was performed to determine the expression patterns of DEGs using R package gplots. Principle component analysis (PCA) were also performed using function princomp() and visualized using scatterplot3d package. R 3.3.1 was used for data manipulation and visualization.

### WGCNA

WGCNA is a biology method to identify genes with similar expression variation trend across different samples. Samples clustering were performed to demonstrate the relationship between expression profile and clinical traits. After raw data preprocessing, weighted gene co-expressison analysis were performed to identify significant gene modules related to DN according to a previously described algorithm^32^. Probe sets were first filtered based on the variance of expression value across all samples. Probe sets with duplicated gene symbols were also removed based on expression variance. Totally 8450 genes were finally selected. The R package WGCNA^23^ was applied for this analysis. Briefly, Person’s correlation coefficients were calculated for selected genes in a pairwise manner yielding a similarity matrix (Sij). The matrix was transformed into an adjacency matrix (aij) using a power function using formula aij = Power (Sij, β) ≡ |Sij| β. Average linkage hierarchical clustering was then performed to identify modules of densely interconnected genes. Genes that were not assigned to specific modules were assigned the color grey. Next, genes within significantly related modules were submitted to online tool DAVID for functional enrichment analysis. All data manipulations were carried out in R 3.3.1.

### Hub gene screening and CNC (coding-non-coding network) construction

Genes within significant related modules were ranked using formula $\sum _{{\rm{s}}\in {\rm{V}}}{{\rm{p}}}_{{\rm{s}}}({\rm{v}})$, in which let T_s be a shortest path tree rooted at s. p_s (v) = 1 if more than |V(T_s)|/4 paths from s to other vertices in T_s meet at the vertex v; otherwise p_s (v) = 0. The top 20 genes were considered as hub genes. CNC network of related modules was constructed using WGCNA output and visualized using Cytoscape version 3.5^33^.

### Validation of DELs (differential expressed lncRNAs)

To validate DELs discovered in the training data set, two data series GSE47183 and GSE47184 were used. After batch effects removal, the expression of important lncRNAs discovered above (DELs in training data set and hub lncRNAs in CNC network) were validated in the two validation sets.

### Validation of DEGs in mice

To validate the DEGs identified in human, differential expression analysis was carried out in two data series in mouse (Using the same method above). Finally, 172 recurrent DEGs were filtered. To further explore the similarity of DEGs identified in human and mouse, functional enrichment analysis was also performed with those 172 DEGs.

### Cell culture and stimulation

HPCs and HMCs were generous gift of Prof. Fan Yi from Shandong University of China. Cells were maintained in DMEM (glucose concentration was 5.6 mM) with 10% fetal bovine serum at 37 °C with 5% CO2. Cells were passaged when they were at 80% convergence. High glucose (final concentration of 40 mM for 24 h) was used as a stimulator while mannitol of the same concentration was added as osmolality control.

### RNA extranction and qPCR

Cells were collected and total RNA were extracted using TRIzol reagent (Invitrogen, Carlsbad, CA, USA). Reverse transcription of mRNA was carried out using HiScript Q RT SuperMix for qPCR (Vazyme, Nanjing, China) according to the manufacturer’s instructions. Relative quantity of lncRNAs or mRNAs was measured by SYBR Green PCR assays by AceQTM qPCR SYBR Green Master Mix (Novland BioPharma, Shanghai, China). GAPDH was used as internal control. Primers were synthesized by GenePharma (Shanghai, China). Primer sequences: NR_130134.1: Forward 5′-TTGCATCCCAGCCCTATTCCTG-3′ Reverse 5′-GCACCCTTGACATTTCCTGTACC-3′; NR_038335.1:Forward 5′-GCTTCCCACACTTCTGACTCG-3′ Reverse 5′-TCAAAGAGCTTCACGACCACC-3′; NR_029395.1: Forward 5′-GGACCCAGCTCACCGTTTTAG-3′ Reverse 5′-CTCCCGTTTACTCCCCGCTA-3′; CD163: Forward 5′-GGGCTAATTCCAGTGCAGGT-3′ Reverse 5′-GCTGACTCATTCCCACGACA-3′; HOPX:Forward 5′-CCGAGGAGGAGACCCAGAAA-3′ Reverse 5′-TGTGACGGATCTGCACTCTG-3′; TDO2:Forward 5′-TCCTCAGGCTATCACTACCTGC-3′ Reverse 5′-ATCTTCGGTATCCAGTGTCGG-3′; CASP1: Forward 5′-ATGCCTGTTCCTGTGATGTGG-3′ Reverse 5′-GGCATCTGCGCTCTACCATC-3′; PTPRC: Forward 5′-TTCTTAGGGACACGGCTGAC-3′ Reverse 5′-TTAAGGTAGGCATCAGTGGGGG-3′; COL6A3: Forward 5′-CAGTTCCCTGTTGTCCGTGA-3′ Reverse 5′-CTGTACTGAGCCACCGCAAT-3′; CST6: Forward 5′-TCCTGACGATGGAGATGGGG-3′ Reverse 5′-GACCTCAAAGTCACAGCGCA-3′; CELF2: Forward 5′-AGCACCAATGCAAACCCTCT-3′ Reverse 5′-CAGAGTCCCGAGAGAGGTCA-3′; KRT7:Forward 5′-TGGAGTGGGAGCCGTGAATA-3′ Reverse 5′-TGCGGTCCGGATGGAATAAG-3′; PYCARD: Forward 5′-TCAGTTTCACACCAGCCTGGAAC-3′ Reverse 5′-AGGTAGGACTGGGACTCCCTTAGG-3′. Data were represented as means ± standard deviation (SD). One-way analysis of variance (ANOVA) followed by Duncan’s multiple range test were used to check the significance of the differences in mean values using Graphpad Prism 5.0 (CA, USA).

### Gene interference

Specific siRNAs targeting lncRNAs were synthesized by GenePharma (Shanghai, China). The sequences are: NR_130134.1 5′-CCAGGUGUCGAUAAGUAAUTT-3′, NR_029395.1 5′-GGGCACCUUAGACUCAGAATT-3′. Lipofectamine 3000 (Invitrogen, Carlsbad, CA, USA) was used for transfection.

### Fluorescence *in situ* hybridization

Location and expression of lncRNAs were visualized using Fluorescence *in situ* hybridization (FISH) which is performed according to previous description^34^. The probe and kit for FISH was purchased from Ribobio (Guangzhou, China). Generally, cultured cells were fixed in paraformaldehyde for 10 min and incubated in Triton X-100 for 5 min. Slides were blocked using pre-hybridization buffer. Probes were added at 5 nM concentration. Images were captured using Fluorescence microscope (Leica, German) and gathered in Photoshop CS6 (Adobe, USA).

## Supplementary information

Datasets 1

## Data Availability

The datasets generated during and/or analyzed during the current study are available in the GEO database (https://www.ncbi.nlm.nih.gov/geo/).
