# Supplementary material for: Identification of key lncRNAs contributing to diabetic nephropathy by gene co-expression network analysis
Source: Sci Rep. 2019 Mar 1;9:3328. doi: 10.1038/s41598-019-39298-9 (PMC6397236; doi:10.1038/s41598-019-39298-9)
Supplement: Supplementary file 1 — Datasets 1 [file 41598_2019_39298_MOESM1_ESM.pdf]

Identification of key lncRNAs contributing to diabetic nephropathy by gene co-repression network analysis

Jin Shang, Shuai Wang, Yumin Jiang, Yiqi Duan, Genyang Cheng, Dong Liu, Jing Xiao, Zhanzheng Zhao

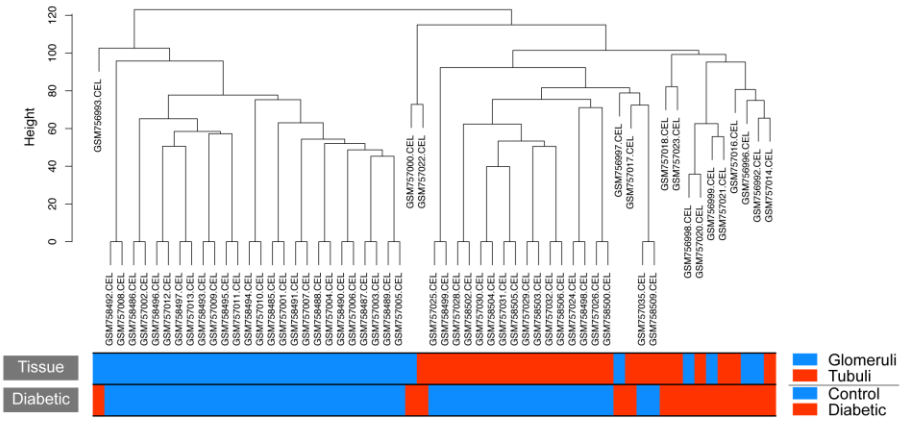

Supplementary Figure S1. Hierarchical clustering of samples in training dataset. Groups of different samples were labeled in the below.

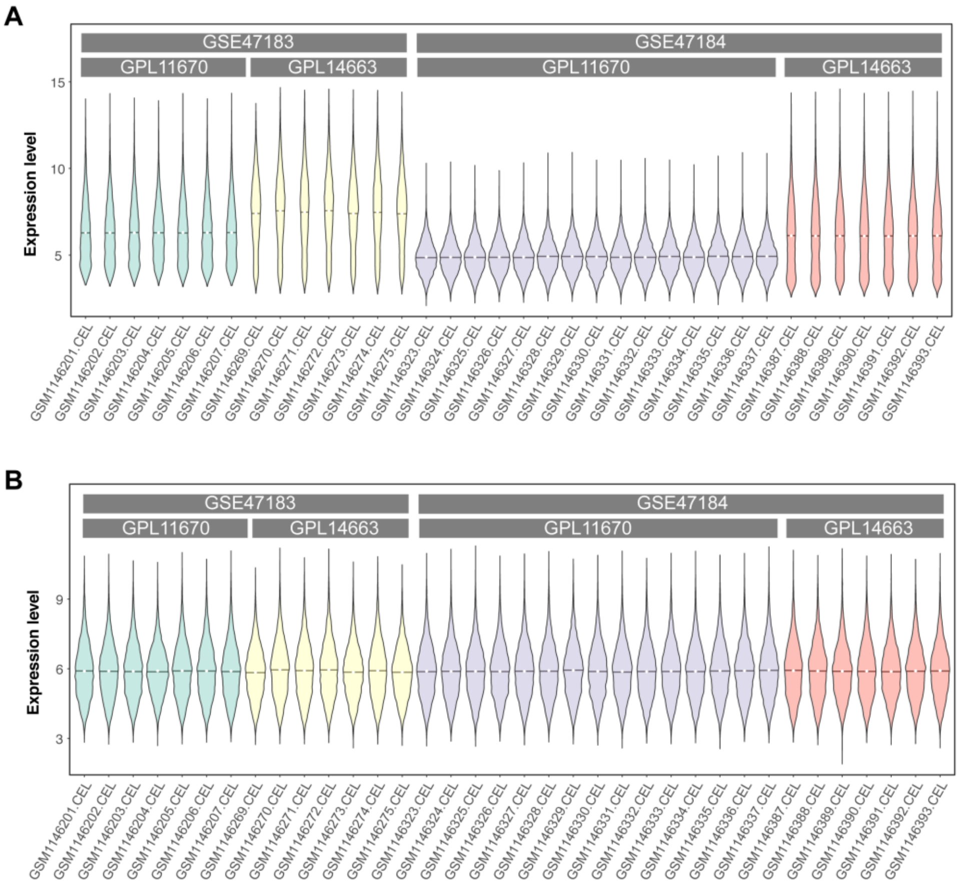

Supplementary Figure S2. Box plots of expression values within all samples. (A) Expression of all samples before batch effects removal. (B) Expression values of all samples after batch effects removal. Y axis corresponds to normalized expression values.

Supplementary Table S1. Differential expressed lncRNAs.

					TD vs TC		GD vs GC	
**RefSeq Accession**	**NOCODE Accession**	**Gene Symbol**	**CNCI Score**	**Chromosome Location**	**Fold change**	**P value**	**Fold change**	**P value**
NR_029395.1	NONHSAT245052.1	IGLL3P	-0.08	chr22:25,318,257-25,320,227	17.18	3.33E-05	5.81	1.25E-02
NR_024448.2	NONHSAT083925.2	GUSBP11	-0.10	chr22:23,638,487-23,717,423	6.48	9.42E-04	4.30	1.52E-02
NR_038335.1	NONHSAT215593.1	CLU	-0.01	chr8:27,596,917-27,611,437	4.75	2.82E-06	3.82	5.42E-05
NR_045494.1	NONHSAT215593.1	CLU	-0.01	chr8:27,596,917-27,611,751	4.75	2.82E-06	3.82	5.42E-05
NR_130134.1	NA	ISG20	-0.02	chr5:35,856,849-35,879,603	3.83	6.39E-05	2.33	1.52E-02
NR_026691.1	NONHSAT160800.1	IL10RA	-0.06	chr11:117,986,391-118,001,483	3.41	3.72E-06	3.20	1.10E-05
NR_033390.1	NONRATT000579.2	TYROBP	-0.03	chr19:35,904,401-35,908,309	3.35	1.37E-05	2.67	4.36E-04

NR_029467.1	NONHSAT007472.2	SELL	-0.01	chr1:169,690,665-169,708,856	3.22	7.16E-05	2.46	2.96E-03
NR_120485.1	NONHSAT171153.1	IL7R	-0.08	chr15:88,635,637-88,656,344	2.33	3.45E-04	3.45	7.28E-07
NR_109871.1	NONMMUT004435.2	TRAF3IP3	-0.06	chr1:209,756,032-209,779,657	2.11	3.27E-02	3.55	8.15E-04

Supplementary Table S2. DEGs identified in human diabetic nephropathy

RefSeq Accession	Gene Symbol	GenBank ID	GD - GC		TD - TC	
			logFC	adj.P.Val	logFC	adj.P.Val
NM_000064.3	C3	718	2.99	4.81E-06	3.97	7.84E-09
NM_000089.3	COL1A2	1278	2.24	5.86E-07	2.63	1.75E-08
NM_000211.4	ITGB2	3689	1.51	3.27E-06	1.81	6.40E-08
NM_000358.2	TGFBI	7045	1.46	3.48E-04	1.77	1.30E-05
NM_000434.3	NEU1	4758	1.01	3.37E-03	1.15	4.59E-04
NM_000491.4	C1QB	713	2.26	2.48E-07	2.31	2.08E-07
NM_000560.3	CD53	963	1.86	2.30E-07	2.18	5.86E-09
NM_000582.2	SPP1	6696	1.15	1.34E-03	1.05	1.90E-03
NM_000587.3	C7	730	2.13	9.50E-06	1.63	3.65E-04
NM_000596.2	IGFBP1	3484	-2.02	1.72E-03	-1.17	4.91E-02
NM_000636.3	SOD2	6648	1.14	1.69E-05	1.49	6.26E-08
NM_000655.4	SELL	6402	1.30	2.96E-03	1.69	7.16E-05
NM_000689.4	ALDH1A1	216	1.32	1.74E-02	1.19	1.91E-02
NM_000693.3	ALDH1A3	220	1.05	7.92E-06	1.03	9.93E-06
NM_000732.4	CD3D	915	1.73	1.93E-05	1.22	1.37E-03
NM_000978.3	RPL23	9349	1.40	7.51E-03	1.13	1.87E-02
NM_001002029.3	C4B	721	1.33	1.88E-02	1.23	1.76E-02
NM_001002033.2	HN1	51155	1.24	3.65E-06	1.05	5.20E-05
NM_001002294.2	FMO3	2328	1.03	2.51E-03	1.29	8.85E-05
NM_001003927.2	EVI2A	2123	2.01	1.74E-05	2.83	9.40E-09
NM_001008389.2	UMOD	7369	-1.56	1.94E-02	-1.63	7.69E-03
NM_001008540.2	CXCR4	7852	1.17	9.38E-04	1.19	4.43E-04
NM_001024465.2	SOD2	6648	1.44	4.92E-06	1.78	6.05E-08
NM_001024466.2	SOD2	6648	1.42	6.21E-06	1.78	5.82E-08
NM_001034.3	RRM2	6241	1.58	1.86E-06	1.53	3.54E-06
NM_001040033.1	CD53	963	1.86	2.30E-07	2.18	5.86E-09
NM_001040058.1	SPP1	6696	1.15	1.34E-03	1.05	1.90E-03
NM_001040060.1	SPP1	6696	1.15	1.34E-03	1.05	1.90E-03
NM_001040651.1	CD3D	915	2.20	8.55E-06	1.69	3.26E-04
NM_001077654.2	TNFAIP8	25816	1.30	2.72E-04	2.23	4.58E-09
NM_001080494.3	TTC39A	22996	1.16	5.48E-07	1.10	1.99E-06
NM_001081455.1	P2RY14	9934	1.43	1.38E-04	1.15	1.17E-03
NM_001085.4	SERPINA3	12	2.55	2.26E-06	2.80	3.53E-07
NM_001098200.1	GPR18	2841	1.36	2.53E-04	1.12	1.37E-03

NM_001100431.1	VSIG4	11326	1.73	4.55E-08	1.32	1.79E-05
NM_001102.3	ACTN1	87	1.21	1.17E-04	1.49	2.33E-06
NM_001122951.2	ACKR1	2532	1.15	7.79E-04	1.44	2.16E-05
NM_001123041.2	CCR2	729230	1.54	3.97E-04	2.50	4.70E-08
NM_001126336.2	VCAN	1462	1.18	6.85E-04	2.27	1.09E-09
NM_001127491.2	ITGB2	3689	1.51	3.27E-06	1.81	6.40E-08
NM_001130004.1	ACTN1	87	1.21	1.17E-04	1.49	2.33E-06
NM_001130005.1	ACTN1	87	1.23	1.24E-04	1.55	1.70E-06
NM_001130415.1	APOLD1	81575	-2.04	1.32E-04	-1.44	3.80E-03
NM_001130715.1	PLAC8	51316	1.06	3.08E-03	1.14	8.35E-04
NM_001130716.1	PLAC8	51316	1.29	9.40E-04	1.47	1.06E-04
NM_001143782.1	FKBP11	51303	1.00	1.50E-02	1.50	1.47E-04
NM_001144832.2	TTC39A	22996	1.16	5.48E-07	1.10	1.99E-06
NM_001145459.1	HOPX	84525	1.09	3.23E-02	2.49	8.99E-07
NM_001145460.1	HOPX	84525	1.09	3.23E-02	2.49	8.99E-07
NM_001146590.1	ETNPPL	64850	-1.38	1.84E-02	-2.24	6.91E-05
NM_001146627.1	ETNPPL	64850	-1.38	1.84E-02	-2.24	6.91E-05
NM_001164097.1	VCAN	1462	1.18	6.85E-04	2.27	1.09E-09
NM_001164098.1	VCAN	1462	1.18	6.85E-04	2.27	1.09E-09
NM_001165931.1	RRM2	6241	1.58	1.86E-06	1.53	3.54E-06
NM_001173514.1	TYROBP	7305	1.42	4.36E-04	1.74	1.37E-05
NM_001173515.1	TYROBP	7305	1.42	4.36E-04	1.74	1.37E-05
NM_001177660.2	HABP2	3026	1.32	1.03E-03	1.14	2.66E-03
NM_001184830.1	VSIG4	11326	1.73	4.55E-08	1.32	1.79E-05
NM_001184831.1	VSIG4	11326	1.73	4.55E-08	1.32	1.79E-05
NM_001193289.1	APOBEC3A_B	100913187	1.11	5.72E-04	1.60	1.16E-06
NM_001193333.2	CORO1A	11151	1.64	1.04E-05	1.58	1.72E-05
NM_001199149.1	LTF	4057	3.34	2.54E-07	4.73	3.36E-11
NM_001199739.1	CTSS	1520	1.03	1.55E-04	1.58	2.73E-08
NM_001207024.1	M6PR	4074	1.01	3.76E-03	1.27	1.54E-04
NM_001242823.2	C4B_2	100293534	1.33	1.88E-02	1.23	1.76E-02
NM_001243266.1	MS4A4A	51338	2.62	3.35E-06	3.39	9.89E-09
NM_001243759.1	USP2	9099	-1.22	1.80E-02	-2.06	4.01E-05
NM_001243962.1	HLA-DQB1	3119	1.39	5.28E-06	1.81	1.51E-08
NM_001247999.1	MS4A6A	64231	2.02	3.10E-08	2.14	1.08E-08
NM_001251829.1	SPP1	6696	1.15	1.34E-03	1.05	1.90E-03
NM_001251830.1	SPP1	6696	1.15	1.34E-03	1.05	1.90E-03
NM_001252204.1	C4A	720	1.33	1.88E-02	1.23	1.76E-02
NM_001255.2	CDC20	991	1.25	2.73E-06	1.05	4.81E-05
NM_001256030.1	CD48	962	1.29	1.29E-06	1.23	3.38E-06
NM_001256139.1	CAPG	822	1.46	1.43E-04	1.16	1.26E-03
NM_001256140.1	CAPG	822	1.36	8.63E-04	1.18	2.06E-03
NM_001257403.1	VSIG4	11326	2.11	7.54E-08	1.90	1.17E-06
NM_001270411.1	APOBEC3B	9582	1.01	2.37E-04	1.20	1.30E-05

NM_001271606.1	BASP1	10409	1.04	4.29E-02	1.13	1.57E-02
NM_001278614.1	UMOD	7369	-1.56	1.94E-02	-1.63	7.69E-03
NM_001278736.1	CCL5	6352	1.80	2.56E-04	1.84	1.11E-04
NM_001282431.1	ARL4C	10123	1.65	2.42E-06	2.15	5.33E-09
NM_001286813.1	TNFAIP8	25816	1.30	2.72E-04	2.23	4.58E-09
NM_001286814.1	TNFAIP8	25816	1.30	2.72E-04	2.23	4.58E-09
NM_001286815.1	TNFAIP8	25816	1.30	2.72E-04	2.23	4.58E-09
NM_001286817.1	TNFAIP8	25816	1.30	2.72E-04	2.23	4.58E-09
NM_001287754.1	TRAF3IP3	80342	1.83	8.15E-04	1.08	3.27E-02
NM_001288609.1	HN1	51155	1.38	7.94E-06	1.21	5.41E-05
NM_001288610.1	HN1	51155	1.38	7.94E-06	1.21	5.41E-05
NM_001293815.1	ALDH1A3	220	1.05	7.92E-06	1.03	9.93E-06
NM_001297663.1	TTC39A	22996	1.16	5.48E-07	1.10	1.99E-06
NM_001297664.1	TTC39A	22996	1.16	5.48E-07	1.10	1.99E-06
NM_001297665.1	TTC39A	22996	1.16	5.48E-07	1.10	1.99E-06
NM_001297666.1	TTC39A	22996	1.16	5.48E-07	1.10	1.99E-06
NM_001297667.1	TTC39A	22996	1.16	5.48E-07	1.10	1.99E-06
NM_001303233.1	ISG20	3669	1.03	1.41E-02	1.24	1.52E-03
NM_001303234.1	ISG20	3669	1.03	1.41E-02	1.24	1.52E-03
NM_001303235.1	ISG20	3669	1.22	1.52E-02	1.94	6.39E-05
NM_001303236.1	ISG20	3669	1.12	1.17E-02	1.47	4.72E-04
NM_001303237.1	ISG20	3669	1.16	9.47E-03	1.50	3.72E-04
NM_001303238.1	ITGB2	3689	1.51	3.27E-06	1.81	6.40E-08
NM_001306129.1	FN1	2335	1.23	7.92E-06	1.43	3.53E-07
NM_001306130.1	FN1	2335	1.23	7.92E-06	1.43	3.53E-07
NM_001306131.1	FN1	2335	1.23	7.92E-06	1.43	3.53E-07
NM_001306132.1	FN1	2335	1.23	7.92E-06	1.43	3.53E-07
NM_001311.4	CRIP1	1396	1.32	6.47E-03	1.54	7.45E-04
NM_001319173.1	FMO3	2328	1.03	2.51E-03	1.29	8.85E-05
NM_001319174.1	FMO3	2328	1.03	2.51E-03	1.29	8.85E-05
NM_001320638.1	CD53	963	1.86	2.30E-07	2.18	5.86E-09
NM_001320732.1	CAPG	822	1.46	1.43E-04	1.16	1.26E-03
NM_001320733.1	CAPG	822	1.46	1.43E-04	1.16	1.26E-03
NM_001320734.1	CAPG	822	1.60	1.10E-04	1.07	5.28E-03
NM_001321121.1	LTF	4057	3.34	2.54E-07	4.73	3.36E-11
NM_001321122.1	LTF	4057	3.34	2.54E-07	4.73	3.36E-11
NM_001322814.1	SOD2	6648	1.13	3.47E-05	1.49	1.40E-07
NM_001322815.1	SOD2	6648	1.13	3.92E-05	1.37	9.05E-07
NM_001322817.1	SOD2	6648	1.44	4.92E-06	1.78	6.05E-08
NM_001322819.1	SOD2	6648	1.14	1.69E-05	1.49	6.26E-08
NM_001322820.1	SOD2	6648	1.14	1.69E-05	1.49	6.26E-08
NM_001323.3	CST6	1474	1.06	1.25E-03	1.16	2.36E-04
NM_001326317.1	CELF2	10659	1.05	5.83E-03	1.36	1.93E-04
NM_001326318.1	CELF2	10659	1.05	5.83E-03	1.36	1.93E-04

NM_001326319.1	CELF2	10659	1.05	5.83E-03	1.36	1.93E-04
NM_001326320.1	CELF2	10659	1.05	5.83E-03	1.36	1.93E-04
NM_001326321.1	CELF2	10659	1.05	5.83E-03	1.36	1.93E-04
NM_001326323.1	CELF2	10659	1.05	5.83E-03	1.36	1.93E-04
NM_001326324.1	CELF2	10659	1.05	5.83E-03	1.36	1.93E-04
NM_001326325.1	CELF2	10659	1.05	5.83E-03	1.36	1.93E-04
NM_001326326.1	CELF2	10659	1.05	5.83E-03	1.36	1.93E-04
NM_001326327.1	CELF2	10659	1.05	5.83E-03	1.36	1.93E-04
NM_001326328.1	CELF2	10659	1.05	5.83E-03	1.36	1.93E-04
NM_001326329.1	CELF2	10659	1.05	5.83E-03	1.36	1.93E-04
NM_001326330.1	CELF2	10659	1.05	5.83E-03	1.36	1.93E-04
NM_001326331.1	CELF2	10659	1.05	5.83E-03	1.36	1.93E-04
NM_001326332.1	CELF2	10659	1.05	5.83E-03	1.36	1.93E-04
NM_001326333.1	CELF2	10659	1.05	5.83E-03	1.36	1.93E-04
NM_001326334.1	CELF2	10659	1.05	5.83E-03	1.36	1.93E-04
NM_001326335.1	CELF2	10659	1.05	5.83E-03	1.36	1.93E-04
NM_001326336.1	CELF2	10659	1.05	5.83E-03	1.36	1.93E-04
NM_001326337.1	CELF2	10659	1.05	5.83E-03	1.36	1.93E-04
NM_001326338.1	CELF2	10659	1.05	5.83E-03	1.36	1.93E-04
NM_001326339.1	CELF2	10659	1.05	5.83E-03	1.36	1.93E-04
NM_001326340.1	CELF2	10659	1.05	5.83E-03	1.36	1.93E-04
NM_001326341.1	CELF2	10659	1.05	5.83E-03	1.36	1.93E-04
NM_001326342.1	CELF2	10659	1.05	5.83E-03	1.36	1.93E-04
NM_001326343.1	CELF2	10659	1.05	5.83E-03	1.36	1.93E-04
NM_001326344.1	CELF2	10659	1.05	5.83E-03	1.36	1.93E-04
NM_001326345.1	CELF2	10659	1.05	5.83E-03	1.36	1.93E-04
NM_001326346.1	CELF2	10659	1.05	5.83E-03	1.36	1.93E-04
NM_001326347.1	CELF2	10659	1.05	5.83E-03	1.36	1.93E-04
NM_001326348.1	CELF2	10659	1.05	5.83E-03	1.36	1.93E-04
NM_001326349.1	CELF2	10659	1.05	5.83E-03	1.36	1.93E-04
NM_001328609.1	CD2	914	1.58	2.61E-06	1.33	4.24E-05
NM_001329903.1	SERPINF1	5176	1.55	2.44E-04	1.48	2.42E-04
NM_001329904.1	SERPINF1	5176	1.55	2.44E-04	1.48	2.42E-04
NM_001329905.1	SERPINF1	5176	1.55	2.44E-04	1.48	2.42E-04
NM_001331031.1	ETNPPL	64850	-1.38	1.84E-02	-2.24	6.91E-05
NM_001331032.1	ETNPPL	64850	-1.38	1.84E-02	-2.24	6.91E-05
NM_001331033.1	ETNPPL	64850	-1.38	1.84E-02	-2.24	6.91E-05
NM_001347465.1	C1QA	712	2.05	1.14E-07	1.78	3.17E-06
NM_001347466.1	C1QA	712	2.05	1.14E-07	1.78	3.17E-06
NM_001347828.1	PDGFRA	5156	1.21	7.51E-03	1.65	1.35E-04
NM_001347829.1	PDGFRA	5156	1.21	7.51E-03	1.65	1.35E-04
NM_001347830.1	PDGFRA	5156	1.21	7.51E-03	1.65	1.35E-04
NM_001348056.1	CXCR4	7852	1.17	9.38E-04	1.19	4.43E-04
NM_001348059.1	CXCR4	7852	1.17	9.38E-04	1.19	4.43E-04

NM_001348060.1	CXCR4	7852	1.17	9.38E-04	1.19	4.43E-04
NM_001558.3	IL10RA	3587	1.68	1.10E-05	1.77	3.72E-06
NM_001710.5	CFB	629	1.79	4.77E-05	2.37	1.78E-07
NM_001747.3	CAPG	822	1.46	1.43E-04	1.16	1.26E-03
NM_001765.2	CD1C	911	1.23	3.83E-08	1.24	6.10E-08
NM_001767.4	CD2	914	1.58	2.61E-06	1.33	4.24E-05
NM_001778.3	CD48	962	1.97	4.87E-07	2.19	6.10E-08
NM_001803.2	CD52	1043	2.10	6.89E-06	1.58	3.67E-04
NM_001831.3	CLU	1191	1.93	5.42E-05	2.25	2.82E-06
NM_001855.4	COL15A1	1306	1.19	1.95E-03	1.92	8.99E-07
NM_001870.3	CPA3	1359	2.09	4.16E-06	2.72	1.12E-08
NM_002001.3	FCER1A	2205	1.23	1.24E-06	1.52	9.58E-09
NM_002010.2	FGF9	2254	-1.66	1.87E-07	-1.15	1.47E-04
NM_002026.3	FN1	2335	1.23	7.92E-06	1.43	3.53E-07
NM_002036.3	ACKR1	2532	1.15	7.79E-04	1.44	2.16E-05
NM_002104.2	GZMK	3003	1.85	1.76E-05	1.50	2.71E-04
NM_002160.3	TNC	3371	1.54	2.36E-04	3.07	3.55E-11
NM_002163.2	IRF8	3394	1.59	1.74E-05	1.88	5.95E-07
NM_002185.3	IL7R	3575	1.78	7.28E-07	1.22	3.45E-04
NM_002201.5	ISG20	3669	1.03	1.41E-02	1.24	1.52E-03
NM_002341.1	LTB	4050	1.86	3.83E-05	1.32	1.86E-03
NM_002343.5	LTF	4057	3.34	2.54E-07	4.73	3.36E-11
NM_002345.3	LUM	4060	2.66	3.17E-06	2.40	1.90E-05
NM_002423.4	MMP7	4316	2.47	5.28E-06	4.39	4.69E-12
NM_002438.3	MRC1	4360	1.48	1.10E-04	1.79	2.85E-06
NM_002615.6	SERPINF1	5176	1.55	2.44E-04	1.48	2.42E-04
NM_002738.6	PRKCB	5579	1.04	1.01E-03	1.08	3.54E-04
NM_002838.4	PTPRC	5788	1.40	4.39E-04	1.93	1.74E-06
NM_002888.3	RARRES1	5918	1.40	6.42E-06	1.09	2.37E-04
NM_002909.4	REG1A	5967	1.67	1.14E-04	1.44	4.75E-04
NM_002985.2	CCL5	6352	1.80	2.56E-04	1.84	1.11E-04
NM_002993.3	CXCL6	6372	2.38	3.20E-06	4.23	2.39E-12
NM_003107.2	SOX4	6659	1.18	2.42E-04	1.34	2.31E-05
NM_003182.2	TAC1	6863	1.76	2.04E-03	2.92	5.38E-07
NM_003247.3	THBS2	7058	2.05	1.79E-06	2.17	5.82E-07
NM_003263.3	TLR1	7096	1.01	1.00E-04	1.25	1.74E-06
NM_003332.3	TYROBP	7305	1.56	1.06E-04	1.76	1.08E-05
NM_003361.3	UMOD	7369	-1.56	1.94E-02	-1.63	7.69E-03
NM_003467.2	CXCR4	7852	1.17	9.38E-04	1.19	4.43E-04
NM_004079.4	CTSS	1520	1.03	1.55E-04	1.58	2.73E-08
NM_004132.4	HABP2	3026	1.32	1.03E-03	1.14	2.66E-03
NM_004205.4	USP2	9099	-1.22	1.80E-02	-2.06	4.01E-05
NM_004244.5	CD163	9332	1.90	1.22E-07	2.11	1.45E-08
NM_004271.3	LY86	9450	1.15	6.17E-05	1.20	2.26E-05

NM_004288.4	CYTIP	9595	1.20	6.71E-03	1.30	1.70E-03
NM_004369.3	COL6A3	1293	2.34	4.92E-06	2.61	4.93E-07
NM_004385.4	VCAN	1462	1.18	6.85E-04	2.27	1.09E-09
NM_004585.4	RARRES3	5920	1.43	2.66E-05	1.28	1.04E-04
NM_004900.4	APOBEC3B	9582	1.01	2.37E-04	1.20	1.30E-05
NM_004946.2	DOCK2	1794	1.24	1.49E-05	1.09	8.10E-05
NM_004951.4	GPR183	1880	1.41	2.86E-04	1.24	7.41E-04
NM_005022.3	PFN1	5216	1.30	7.77E-04	1.30	4.23E-04
NM_005292.3	GPR18	2841	1.36	2.53E-04	1.12	1.37E-03
NM_005556.3	KRT7	3855	1.24	6.01E-04	1.13	9.79E-04
NM_005615.4	RNASE6	6039	1.43	3.50E-07	1.57	6.10E-08
NM_005651.3	TDO2	6999	1.50	1.46E-05	1.12	6.08E-04
NM_005727.3	TSPAN1	10103	1.40	5.42E-03	1.26	6.70E-03
NM_005737.3	ARL4C	10123	1.65	2.42E-06	2.15	5.33E-09
NM_005849.3	IGSF6	10261	1.08	1.83E-03	1.38	4.90E-05
NM_006098.4	RACK1	10399	1.13	1.61E-03	1.08	1.38E-03
NM_006103.3	WFDC2	10406	2.18	1.35E-05	2.22	9.08E-06
NM_006144.3	GZMA	3001	2.00	2.73E-05	2.30	1.77E-06
NM_006169.2	NNMT	4837	2.59	2.07E-07	3.22	1.08E-09
NM_006206.5	PDGFRA	5156	1.21	7.51E-03	1.65	1.35E-04
NM_006274.2	CCL19	6363	1.97	2.31E-04	2.04	8.44E-05
NM_006317.4	BASP1	10409	1.04	4.29E-02	1.13	1.57E-02
NM_006398.3	UBD	10537	1.28	2.90E-03	1.70	5.08E-05
NM_006408.3	AGR2	10551	1.29	7.79E-04	1.98	4.80E-07
NM_006495.3	EVI2B	2124	1.66	9.96E-04	2.55	7.31E-07
NM_006762.2	LAPTM5	7805	1.52	4.87E-05	2.14	4.71E-08
NM_006894.5	FMO3	2328	1.03	2.51E-03	1.29	8.85E-05
NM_007074.3	CORO1A	11151	1.64	1.04E-05	1.58	1.72E-05
NM_007268.2	VSIG4	11326	1.73	4.55E-08	1.32	1.79E-05
NM_007293.2	C4A	720	1.33	1.88E-02	1.23	1.76E-02
NM_009588.1	LTB	4050	1.86	3.83E-05	1.32	1.86E-03
NM_013258.4	PYCARD	29108	1.64	8.59E-04	1.86	1.01E-04
NM_013996.2	TAC1	6863	1.76	2.04E-03	2.92	5.38E-07
NM_013997.2	TAC1	6863	1.76	2.04E-03	2.92	5.38E-07
NM_013998.2	TAC1	6863	1.76	2.04E-03	2.92	5.38E-07
NM_014210.3	EVI2A	2123	2.01	1.74E-05	2.83	9.40E-09
NM_014350.3	TNFAIP8	25816	1.30	2.72E-04	2.23	4.58E-09
NM_014879.3	P2RY14	9934	1.43	1.38E-04	1.15	1.17E-03
NM_015529.3	MOXD1	26002	2.02	4.93E-05	2.27	5.25E-06
NM_015675.3	GADD45B	4616	-1.40	2.68E-04	-1.30	4.09E-04
NM_015991.3	C1QA	712	2.05	1.14E-07	1.78	3.17E-06
NM_016562.3	TLR7	51284	1.33	2.44E-04	1.39	7.73E-05
NM_016619.2	PLAC8	51316	1.29	9.40E-04	1.47	1.06E-04
NM_018460.3	ARHGAP15	55843	1.19	4.51E-05	1.10	9.53E-05

NM_022349.3	MS4A6A	64231	2.02	3.10E-08	2.14	1.08E-08
NM_030817.2	APOLD1	81575	-2.04	1.32E-04	-1.44	3.80E-03
NM_031279.3	ETNPPL	64850	-1.38	1.84E-02	-2.24	6.91E-05
NM_032495.5	HOPX	84525	1.09	3.23E-02	2.49	8.99E-07
NM_033295.3	CASP1	834	1.16	2.56E-04	2.37	3.36E-11
NM_057166.4	COL6A3	1293	2.34	4.92E-06	2.61	4.93E-07
NM_057167.3	COL6A3	1293	2.34	4.92E-06	2.61	4.93E-07
NM_080921.3	PTPRC	5788	1.40	4.39E-04	1.93	1.74E-06
NM_139211.4	HOPX	84525	1.09	3.23E-02	2.49	8.99E-07
NM_139212.3	HOPX	84525	1.09	3.23E-02	2.49	8.99E-07
NM_144646.3	JCHAIN	3512	3.02	3.25E-06	4.22	1.09E-09
NM_145182.2	PYCARD	29108	1.64	8.59E-04	1.86	1.01E-04
NM_171997.2	USP2	9099	-1.22	1.80E-02	-2.06	4.01E-05
NM_198125.2	TYROBP	7305	1.56	1.06E-04	1.76	1.08E-05
NM_203416.3	CD163	9332	2.00	2.45E-07	2.18	5.40E-08
NM_206963.1	RARRES1	5918	2.02	1.41E-07	1.75	3.75E-06
NM_212474.2	FN1	2335	1.23	7.92E-06	1.43	3.53E-07
NM_212476.2	FN1	2335	1.23	7.92E-06	1.43	3.53E-07
NM_212478.2	FN1	2335	1.23	7.92E-06	1.43	3.53E-07
NM_212482.2	FN1	2335	1.23	7.92E-06	1.43	3.53E-07
NR_024448.2	GUSBP11	91316	2.11	1.52E-02	2.70	9.42E-04
NR_026691.1	IL10RA	3587	1.68	1.10E-05	1.77	3.72E-06
NR_029395.1	IGLL3P	91353	2.54	1.25E-02	4.10	3.33E-05
NR_029467.1	SELL	6402	1.30	2.96E-03	1.69	7.16E-05
NR_033390.1	TYROBP	7305	1.42	4.36E-04	1.74	1.37E-05
NR_038335.1	CLU	1191	1.93	5.42E-05	2.25	2.82E-06
NR_045494.1	CLU	1191	1.93	5.42E-05	2.25	2.82E-06
NR_109871.1	TRAF3IP3	80342	1.83	8.15E-04	1.08	3.27E-02
NR_120485.1	IL7R	3575	1.78	7.28E-07	1.22	3.45E-04
NR_130134.1	ISG20	3669	1.22	1.52E-02	1.94	6.39E-05
XM_005245625.1	CD48	962	2.65	3.72E-07	3.28	2.45E-09
XM_005246065.1	COL6A3	1293	2.34	4.92E-06	2.61	4.93E-07
XM_005246066.1	COL6A3	1293	2.34	4.92E-06	2.61	4.93E-07
XM_005246402.1	FN1	2335	1.23	7.92E-06	1.43	3.53E-07
XM_005246405.1	FN1	2335	1.23	7.92E-06	1.43	3.53E-07
XM_005246406.1	FN1	2335	1.23	7.92E-06	1.43	3.53E-07
XM_005246409.1	FN1	2335	1.23	7.92E-06	1.43	3.53E-07
XM_005246412.1	FN1	2335	1.23	7.92E-06	1.43	3.53E-07
XM_005246414.1	FN1	2335	1.23	7.92E-06	1.43	3.53E-07
XM_005247686.4	RARRES1	5918	2.01	5.44E-08	1.76	1.57E-06
XM_005247922.3	P2RY14	9934	1.43	1.38E-04	1.15	1.17E-03
XM_005247923.3	P2RY14	9934	1.43	1.38E-04	1.15	1.17E-03
XM_005248299.3	IL7R	3575	1.78	7.28E-07	1.22	3.45E-04
XM_005249051.4	HLA-DQB2	3120	1.09	1.38E-04	1.19	2.37E-05

XM_005249581.4	AGR2	10551	1.29	7.79E-04	1.98	4.80E-07
XM_005251972.3	TNC	3371	1.54	2.36E-04	3.07	3.55E-11
XM_005251973.3	TNC	3371	1.54	2.36E-04	3.07	3.55E-11
XM_005251974.3	TNC	3371	1.54	2.36E-04	3.07	3.55E-11
XM_005251975.3	TNC	3371	1.54	2.36E-04	3.07	3.55E-11
XM_005253274.3	LOC101927562	101927562	-1.15	9.73E-03	-1.04	1.13E-02
XM_005253529.4	CD163	9332	2.04	1.58E-07	2.16	6.10E-08
XM_005254899.2	ISG20	3669	1.03	1.41E-02	1.24	1.52E-03
XM_005257946.3	EVI2B	2124	1.66	9.96E-04	2.55	7.31E-07
XM_005259642.1	VAV1	7409	1.06	5.97E-03	1.11	2.09E-03
XM_005262662.4	TLR1	7096	1.01	1.00E-04	1.25	1.74E-06
XM_005265743.1	PDGFRA	5156	1.21	7.51E-03	1.65	1.35E-04
XM_005271721.4	USP2	9099	-1.22	1.80E-02	-2.06	4.01E-05
XM_005271722.2	USP2	9099	-1.22	1.80E-02	-2.06	4.01E-05
XM_006711472.3	PTPRC	5788	1.40	4.39E-04	1.93	1.74E-06
XM_006711473.3	PTPRC	5788	1.40	4.39E-04	1.93	1.74E-06
XM_006711474.3	PTPRC	5788	1.40	4.39E-04	1.93	1.74E-06
XM_006712253.1	COL6A3	1293	2.34	4.92E-06	2.61	4.93E-07
XM_006714211.2	JCHAIN	3512	3.02	3.25E-06	4.22	1.09E-09
XM_006714212.2	JCHAIN	3512	3.02	3.25E-06	4.22	1.09E-09
XM_006716284.2	CLU	1191	1.99	1.45E-04	2.46	2.87E-06
XM_006717096.3	TNC	3371	1.54	2.36E-04	3.07	3.55E-11
XM_006717097.3	TNC	3371	1.54	2.36E-04	3.07	3.55E-11
XM_006717098.3	TNC	3371	1.54	2.36E-04	3.07	3.55E-11
XM_006717101.3	TNC	3371	1.54	2.36E-04	3.07	3.55E-11
XM_006718661.2	MS4A6A	64231	2.02	3.10E-08	2.14	1.08E-08
XM_006719946.3	GPR18	2841	1.36	2.53E-04	1.12	1.37E-03
XM_006720488.3	ISG20	3669	1.03	1.41E-02	1.24	1.52E-03
XM_006724001.1	ITGB2	3689	1.51	3.27E-06	1.81	6.40E-08
XM_011509345.2	FMO3	2328	1.03	2.51E-03	1.29	8.85E-05
XM_011510018.2	TRAF3IP3	80342	1.89	1.19E-03	1.33	1.33E-02
XM_011510171.1	CD48	962	1.97	4.87E-07	2.19	6.10E-08
XM_011510574.1	COL6A3	1293	2.34	4.92E-06	2.61	4.93E-07
XM_011511479.2	ARHGAP15	55843	1.19	4.51E-05	1.10	9.53E-05
XM_011511483.1	ARHGAP15	55843	1.19	4.51E-05	1.10	9.53E-05
XM_011513340.2	P2RY14	9934	1.43	1.38E-04	1.15	1.17E-03
XM_011513742.2	TLR1	7096	1.01	1.00E-04	1.25	1.74E-06
XM_011513743.2	TLR1	7096	1.01	1.00E-04	1.25	1.74E-06
XM_011513744.2	TLR1	7096	1.01	1.00E-04	1.25	1.74E-06
XM_011513745.2	TLR1	7096	1.01	1.00E-04	1.25	1.74E-06
XM_011518214.2	COL15A1	1306	1.19	1.95E-03	1.92	8.99E-07
XM_011518625.2	TNC	3371	1.54	2.36E-04	3.07	3.55E-11
XM_011518626.2	TNC	3371	1.54	2.36E-04	3.07	3.55E-11
XM_011518628.2	TNC	3371	1.54	2.36E-04	3.07	3.55E-11

XM_011518629.2	TNC	3371	1.54	2.36E-04	3.07	3.55E-11
XM_011521521.2	ISG20	3669	1.22	1.52E-02	1.94	6.39E-05
XM_011531925.1	JCHAIN	3512	3.02	3.25E-06	4.22	1.09E-09
XM_011531926.1	JCHAIN	3512	3.02	3.25E-06	4.22	1.09E-09
XM_011533122.1	CAPG	822	1.46	1.43E-04	1.16	1.26E-03
XM_011533123.1	CAPG	822	1.46	1.43E-04	1.16	1.26E-03
XM_011534996.2	FGF9	2254	-1.66	1.87E-07	-1.15	1.47E-04
XM_011537265.2	ACTN1	87	1.21	1.17E-04	1.49	2.33E-06
XM_011537266.2	ACTN1	87	1.21	2.93E-04	1.54	4.07E-06
XM_011537267.2	ACTN1	87	1.20	2.39E-04	1.50	4.38E-06
XM_011537268.2	ACTN1	87	1.21	2.93E-04	1.54	4.07E-06
XM_011538325.1	KRT7	3855	1.10	9.14E-03	1.48	2.53E-04
XM_011540460.2	TSPAN1	10103	1.36	1.28E-02	1.32	8.71E-03
XM_011542059.2	C1QB	713	2.30	4.05E-08	2.14	3.57E-07
XM_011542098.2	LAPTM5	7805	1.52	4.87E-05	2.14	4.71E-08
XM_011545714.2	CORO1A	11151	1.64	1.04E-05	1.58	1.72E-05
XM_011545934.1	UMOD	7369	-1.56	1.94E-02	-1.63	7.69E-03
XM_011545935.1	UMOD	7369	-1.56	1.94E-02	-1.63	7.69E-03
XM_011545936.1	UMOD	7369	-1.56	1.94E-02	-1.63	7.69E-03
XM_011545937.1	UMOD	7369	-1.56	1.94E-02	-1.63	7.69E-03
XM_011545938.1	UMOD	7369	-1.56	1.94E-02	-1.63	7.69E-03
XM_011545940.1	UMOD	7369	-1.56	1.94E-02	-1.63	7.69E-03
XM_017002867.1	CD48	962	2.39	3.47E-07	2.59	7.02E-08
XM_017003303.1	COL6A3	1293	2.34	4.92E-06	2.61	4.93E-07
XM_017003304.1	COL6A3	1293	2.34	4.92E-06	2.61	4.93E-07
XM_017003692.1	FN1	2335	1.23	7.92E-06	1.43	3.53E-07
XM_017003693.1	FN1	2335	1.23	7.92E-06	1.43	3.53E-07
XM_017003694.1	FN1	2335	1.23	7.92E-06	1.43	3.53E-07
XM_017004499.1	ARHGAP15	55843	1.19	4.51E-05	1.10	9.53E-05
XM_017004500.1	ARHGAP15	55843	1.19	4.51E-05	1.10	9.53E-05
XM_017005386.1	CYTIP	9595	1.20	6.71E-03	1.30	1.70E-03
XM_017007583.1	P2RY14	9934	1.43	1.38E-04	1.15	1.17E-03
XM_017008564.1	SPP1	6696	1.15	1.34E-03	1.05	1.90E-03
XM_017008571.1	TLR1	7096	1.01	1.00E-04	1.25	1.74E-06
XM_017008572.1	TLR1	7096	1.01	1.00E-04	1.25	1.74E-06
XM_017008728.1	HOPX	84525	1.09	3.23E-02	2.49	8.99E-07
XM_017008729.1	HOPX	84525	1.09	3.23E-02	2.49	8.99E-07
XM_017008730.1	HOPX	84525	1.09	3.23E-02	2.49	8.99E-07
XM_017008731.1	HOPX	84525	1.09	3.23E-02	2.49	8.99E-07
XM_017008732.1	HOPX	84525	1.09	3.23E-02	2.49	8.99E-07
XM_017008733.1	HOPX	84525	1.09	3.23E-02	2.49	8.99E-07
XM_017008734.1	HOPX	84525	1.09	3.23E-02	2.49	8.99E-07
XM_017009327.1	TNFAIP8	25816	1.30	2.72E-04	2.23	4.58E-09
XM_017009328.1	TNFAIP8	25816	1.30	2.72E-04	2.23	4.58E-09

XM_017010714.1	MOXD1	26002	2.02	4.93E-05	2.27	5.25E-06
XM_017010715.1	MOXD1	26002	2.02	4.93E-05	2.27	5.25E-06
XM_017014678.1	TNC	3371	1.54	2.36E-04	3.07	3.55E-11
XM_017014679.1	TNC	3371	1.54	2.36E-04	3.07	3.55E-11
XM_017014680.1	TNC	3371	1.54	2.36E-04	3.07	3.55E-11
XM_017014681.1	TNC	3371	1.54	2.36E-04	3.07	3.55E-11
XM_017018124.1	MS4A6A	64231	2.02	3.10E-08	2.14	1.08E-08
XM_017018539.1	USP2	9099	-1.22	1.80E-02	-2.06	4.01E-05
XM_017018543.1	CD3D	915	1.73	1.93E-05	1.22	1.37E-03
XM_017019294.1	KRT7	3855	1.24	6.01E-04	1.13	9.79E-04
XM_017020405.1	GPR183	1880	1.41	2.86E-04	1.24	7.41E-04
XM_017021566.1	RNASE6	6039	1.43	3.50E-07	1.57	6.10E-08
XM_017021567.1	RNASE6	6039	1.43	3.50E-07	1.57	6.10E-08
XM_017021720.1	ACTN1	87	1.21	1.17E-04	1.49	2.33E-06
XM_017021721.1	ACTN1	87	1.21	1.17E-04	1.49	2.33E-06
XM_017021722.1	ACTN1	87	1.21	1.17E-04	1.49	2.33E-06
XM_017021723.1	ACTN1	87	1.21	1.17E-04	1.49	2.33E-06
XM_017021725.1	ACTN1	87	1.21	1.17E-04	1.49	2.33E-06
XM_017021726.1	ACTN1	87	1.21	1.17E-04	1.49	2.33E-06
XM_017021727.1	ACTN1	87	1.21	2.93E-04	1.54	4.07E-06
XM_017021728.1	ACTN1	87	1.21	2.93E-04	1.54	4.07E-06
XM_017022147.1	ISG20	3669	1.12	1.17E-02	1.47	4.72E-04
XM_017022148.1	ISG20	3669	1.16	9.47E-03	1.50	3.72E-04
XM_017022885.1	CORO1A	11151	1.64	1.04E-05	1.58	1.72E-05
XM_017022886.1	CORO1A	11151	1.64	1.04E-05	1.58	1.72E-05
XM_017023199.1	IRF8	3394	1.59	1.74E-05	1.88	5.95E-07
XM_017023641.1	UMOD	7369	-1.56	1.94E-02	-1.63	7.69E-03
XM_017024761.1	PFN1	5216	1.30	1.65E-03	1.33	7.39E-04
XM_017026822.1	GADD45B	4616	-1.40	2.68E-04	-1.30	4.09E-04
XM_017028341.1	ITGB2	3689	1.51	3.27E-06	1.81	6.40E-08
XM_017029251.1	VSIG4	11326	2.11	7.54E-08	1.90	1.17E-06
XM_017030289.1	C4A	720	1.33	1.88E-02	1.23	1.76E-02
XR_001738850.1	ARHGAP15	55843	1.19	4.51E-05	1.10	9.53E-05
XR_001740228.1	RARRES1	5918	2.37	2.45E-09	1.97	4.36E-07
XR_001748699.1	KRT7	3855	1.24	6.01E-04	1.13	9.79E-04
XR_001748700.1	KRT7	3855	1.10	9.14E-03	1.48	2.53E-04
XR_925163.2	TLR1	7096	1.01	1.00E-04	1.25	1.74E-06
XR_925165.1	TLR1	7096	1.01	1.00E-04	1.25	1.74E-06

Supplementary Table S3. Preservation score of modules derived from WGCNA

Module	MedianRank.pres	MedianRank.qual	Zsummary.pres	Zsummary.qual
black	10	14	29	37

blue	19	22	46	41
brown	16	20	49	55
cyan	5	10	20	18
darkgreen	4	15	12	10
darkred	2	3	13	15
gold	23	23	34	-0.62
green	21	21	30	38
greenyellow	18	19	18	16
grey	23	24	5.7	-1.8
grey60	22	12.5	11	11
lightcyan	15	11	13	15
lightgreen	3	4	14	16
lightyellow	11	7.5	12	13
magenta	10	8.5	18	24
midnightblue	8	6	13	16
pink	6	2	21	41
purple	12	18	17	17
red	11	12.5	34	46
royalblue	2	1	15	19
salmon	20	5	13	30
tan	6	8	23	21
turquoise	14	16.5	47	65
yellow	14	16.5	38	50

Supplementary Table S4. Functional enrichment of genes in significant modules

Module	Category	GO Term/Pathway	Gene count	Gene Ratio (%)	P Value	Fold Enrichment
lightcyan	BP	Cytokine secretion	3	21.43	7.09E-03	21.62
lightcyan	BP	Regulation of protein metabolic process	7	50.00	7.73E-03	3.31
lightcyan	BP	Blood vessel development	4	28.57	9.09E-03	8.26
lightcyan	BP	Regulation of cytokine production	4	28.57	9.13E-03	8.24
lightcyan	BP	Negative regulation of protein transport	3	21.43	9.59E-03	18.49
lightcyan	BP	Vasculature development	4	28.57	1.06E-02	7.80
lightcyan	BP	Negative regulation of establishment of protein localization	3	21.43	1.07E-02	17.49
lightcyan	BP	Cytokine production	4	28.57	1.17E-02	7.54
lightcyan	BP	Response to growth factor	4	28.57	1.18E-02	7.50
lightcyan	BP	Regulation of erk1 and erk2 cascade	3	21.43	1.56E-02	14.33
lightcyan	BP	Regulation of response to external stimulus	4	28.57	1.67E-02	6.60
lightcyan	BP	Negative regulation of response to stimulus	5	35.71	1.78E-02	4.34
lightcyan	BP	Regulation of immune effector process	3	21.43	2.57E-02	10.98
lightcyan	BP	Extracellular matrix organization	3	21.43	2.70E-02	10.68

lightcyan	BP	Extracellular structure organization	3	21.43	2.72E-02	10.65
lightcyan	BP	Circulatory system development	4	28.57	3.40E-02	5.03
lightcyan	BP	Cardiovascular system development	4	28.57	3.40E-02	5.03
lightcyan	BP	Positive regulation of cytokine production	3	21.43	3.53E-02	9.24
lightcyan	BP	Cell adhesion	5	35.71	3.64E-02	3.50
lightcyan	BP	Biological adhesion	5	35.71	3.68E-02	3.49
lightcyan	BP	Macrophage activation	2	14.29	3.76E-02	48.54
lightcyan	BP	Symbiosis, encompassing mutualism through parasitism	4	28.57	3.91E-02	4.77
lightcyan	BP	Interspecies interaction between organisms	4	28.57	3.91E-02	4.77
lightcyan	BP	Angiogenesis	3	21.43	4.00E-02	8.64
lightcyan	BP	Negative regulation of multicellular organismal process	4	28.57	4.10E-02	4.68
lightcyan	BP	Positive regulation of fibroblast proliferation	2	14.29	4.21E-02	43.25
lightcyan	BP	Cellular response to chemical stimulus	6	42.86	4.28E-02	2.69
lightcyan	BP	Negative regulation of transport	3	21.43	4.67E-02	7.93
lightcyan	BP	Regulation of signal transduction	6	42.86	4.79E-02	2.62
lightcyan	BP	Secretion	4	28.57	4.97E-02	4.33
lightcyan	CC	Extracellular matrix	6	42.86	2.17E-05	14.74
lightcyan	CC	Extracellular matrix component	4	28.57	9.19E-05	40.68
lightcyan	CC	Proteinaceous extracellular matrix	5	35.71	9.40E-05	18.03
lightcyan	CC	Cytoplasmic, membrane-bounded vesicle	6	42.86	9.47E-04	6.60
lightcyan	CC	Membrane-bounded vesicle	9	64.29	1.16E-03	3.24
lightcyan	CC	Cytoplasmic vesicle	6	42.86	1.37E-03	6.08
lightcyan	CC	Intracellular vesicle	6	42.86	1.38E-03	6.07
lightcyan	CC	Vesicle	9	64.29	1.52E-03	3.12
lightcyan	CC	Extracellular region part	9	64.29	1.90E-03	3.02
lightcyan	CC	Collagen trimer	3	21.43	2.32E-03	38.29
lightcyan	CC	Extracellular space	6	42.86	2.33E-03	5.41
lightcyan	CC	Extracellular region	9	64.29	6.20E-03	2.53
lightcyan	CC	Endomembrane system	8	57.14	9.95E-03	2.69
lightcyan	CC	Secretory granule	3	21.43	2.60E-02	10.91
lightcyan	CC	Secretory vesicle	3	21.43	4.24E-02	8.36
lightcyan	MF	Collagen binding	3	21.43	9.11E-04	60.87
lightcyan	MF	Growth factor binding	3	21.43	3.58E-03	30.43
lightcyan	MF	Transforming growth factor beta binding	2	14.29	1.13E-02	162.32
lightcyan	MF	Calcium ion binding	4	28.57	1.26E-02	7.24
black	BP	Immune response	28	16.37	2.63E-15	7.02
black	BP	Inflammatory response	23	13.45	8.94E-12	6.41
black	BP	Innate immune response	21	12.28	4.05E-09	5.16
black	BP	Cell adhesion	21	12.28	1.22E-08	4.83
black	BP	Chemokine-mediated signaling pathway	10	5.85	2.08E-08	14.87
black	BP	Cell chemotaxis	9	5.26	1.65E-07	14.62

black	BP	Chemotaxis	11	6.43	2.28E-07	9.52
black	BP	Adaptive immune response	11	6.43	1.37E-06	7.85
black	BP	Cell surface receptor signaling pathway	14	8.19	1.97E-06	5.40
black	BP	Leukocyte migration	10	5.85	2.27E-06	8.66
black	BP	Positive regulation of tumor necrosis factor production	7	4.09	4.93E-06	15.73
black	BP	Dendritic cell chemotaxis	5	2.92	1.63E-05	31.06
black	BP	Extracellular matrix organization	11	6.43	1.67E-05	5.93
black	BP	Neutrophil chemotaxis	7	4.09	3.60E-05	11.20
black	BP	Regulation of immune response	10	5.85	4.77E-05	5.93
black	BP	Regulation of cell shape	9	5.26	5.37E-05	6.79
black	BP	Lymphocyte chemotaxis	5	2.92	1.30E-04	18.86
black	BP	Response to lipopolysaccharide	9	5.26	1.63E-04	5.80
black	BP	Defense response to virus	9	5.26	1.69E-04	5.76
black	BP	T cell differentiation	5	2.92	1.71E-04	17.60
black	BP	Cellular response to interferon-gamma	6	3.51	1.95E-04	11.12
black	BP	Lipopolysaccharide-mediated signaling pathway	5	2.92	2.21E-04	16.50
black	BP	Positive regulation of erk1 and erk2 cascade	9	5.26	2.53E-04	5.43
black	BP	Regulation of inflammatory response	6	3.51	3.13E-04	10.06
black	BP	Integrin-mediated signaling pathway	7	4.09	3.44E-04	7.47
black	BP	Negative regulation of viral genome replication	5	2.92	5.30E-04	13.20
black	BP	Signal transduction	24	14.04	5.44E-04	2.18
black	BP	Interferon-gamma-mediated signaling pathway	6	3.51	5.47E-04	8.92
black	BP	Cellular response to interleukin-1	6	3.51	5.47E-04	8.92
black	BP	Cellular response to tumor necrosis factor	7	4.09	6.04E-04	6.72
black	BP	Monocyte chemotaxis	5	2.92	6.39E-04	12.57
black	BP	Cellular response to lipopolysaccharide	7	4.09	6.96E-04	6.54
black	BP	Positive regulation of tyrosine phosphorylation of stat protein	3	1.75	8.64E-04	63.37
black	BP	B cell receptor signaling pathway	5	2.92	1.66E-03	9.78
black	BP	Cellular calcium ion homeostasis	6	3.51	1.85E-03	6.81
black	BP	Humoral immune response	5	2.92	2.02E-03	9.26
black	BP	Positive regulation of t cell proliferation	5	2.92	2.44E-03	8.80
black	BP	Negative regulation of angiogenesis	5	2.92	2.76E-03	8.52
black	BP	Cell-cell signaling	9	5.26	2.82E-03	3.74
black	BP	Positive regulation of t cell migration	3	1.75	3.77E-03	31.68
black	BP	Immunological synapse formation	3	1.75	3.77E-03	31.68
black	BP	Positive regulation of i-kappab kinase/nf-kappab signaling	7	4.09	4.20E-03	4.59
black	BP	Positive regulation of macrophage chemotaxis	3	1.75	4.58E-03	28.80
black	BP	Platelet activation	6	3.51	4.63E-03	5.51
black	BP	Positive regulation of inflammatory response	5	2.92	4.96E-03	7.23

black	BP	Negative regulation of cell adhesion	4	2.34	5.02E-03	11.42
black	BP	Response to hypoxia	7	4.09	5.81E-03	4.30
black	BP	Macrophage chemotaxis	3	1.75	6.41E-03	24.37
black	BP	Cytokine-mediated signaling pathway	6	3.51	7.97E-03	4.84
black	BP	Positive regulation of interleukin-6 production	4	2.34	8.69E-03	9.39
black	BP	Positive regulation of interferon-gamma production	4	2.34	9.23E-03	9.18
black	BP	Skeletal system development	6	3.51	9.57E-03	4.63
black	BP	Positive regulation of monocyte chemotaxis	3	1.75	9.68E-03	19.80
black	BP	Phagocytosis	4	2.34	1.04E-02	8.80
black	BP	Negative regulation of apoptotic process	11	6.43	1.10E-02	2.55
black	BP	Defense response to bacterium	6	3.51	1.20E-02	4.37
black	BP	Positive regulation of t cell activation	3	1.75	1.22E-02	17.60
black	BP	Toll-like receptor 4 signaling pathway	3	1.75	1.22E-02	17.60
black	BP	Positive regulation of cell proliferation	11	6.43	1.28E-02	2.49
black	BP	Platelet degranulation	5	2.92	1.63E-02	5.13
black	BP	Positive regulation of nf-kappab import into nucleus	3	1.75	1.64E-02	15.09
black	BP	Response to ethanol	5	2.92	1.73E-02	5.03
black	BP	Cellular response to cytokine stimulus	3	1.75	1.80E-02	14.40
black	BP	Positive regulation of neutrophil chemotaxis	3	1.75	1.80E-02	14.40
black	BP	Positive regulation of interleukin-1 beta secretion	3	1.75	1.80E-02	14.40
black	BP	Apoptotic process	12	7.02	1.84E-02	2.24
black	BP	Fc receptor mediated stimulatory signaling pathway	2	1.17	1.87E-02	105.61
black	BP	Positive regulation of hematopoietic stem cell migration	2	1.17	1.87E-02	105.61
black	BP	Positive regulation of immune complex clearance by monocytes and macrophages	2	1.17	1.87E-02	105.61
black	BP	Bundle of his cell to purkinje myocyte communication	2	1.17	1.87E-02	105.61
black	BP	Osteoclast differentiation	3	1.75	1.96E-02	13.78
black	BP	Response to virus	5	2.92	2.02E-02	4.80
black	BP	Cellular defense response	4	2.34	2.06E-02	6.81
black	BP	Collagen catabolic process	4	2.34	2.24E-02	6.60
black	BP	G-protein coupled receptor signaling pathway	16	9.36	2.24E-02	1.88
black	BP	Leukocyte cell-cell adhesion	3	1.75	2.29E-02	12.67
black	BP	Positive regulation of angiogenesis	5	2.92	2.33E-02	4.59
black	BP	Positive regulation of apoptotic process	8	4.68	2.36E-02	2.82
black	BP	Tumor necrosis factor-mediated signaling pathway	5	2.92	2.53E-02	4.48
black	BP	Toll-like receptor signaling pathway	3	1.75	2.65E-02	11.73
black	BP	Negative regulation of cysteine-type endopeptidase activity involved in apoptotic process	4	2.34	2.73E-02	6.12

black	BP	Negative regulation of endopeptidase activity	5	2.92	2.75E-02	4.36
black	BP	Myeloid dendritic cell activation involved in immune response	2	1.17	2.80E-02	70.41
black	BP	Positive regulation of type i hypersensitivity	2	1.17	2.80E-02	70.41
black	BP	Cellular response to triacyl bacterial lipopeptide	2	1.17	2.80E-02	70.41
black	BP	TLR2 signaling pathway	2	1.17	2.80E-02	70.41
black	BP	Cellular response to mechanical stimulus	4	2.34	2.93E-02	5.95
black	BP	Positive regulation of phagocytosis	3	1.75	3.03E-02	10.93
black	BP	Cellular response to fibroblast growth factor stimulus	3	1.75	3.22E-02	10.56
black	BP	Negative regulation of cell proliferation	9	5.26	3.39E-02	2.40
black	BP	Regulation of heart contraction	3	1.75	3.42E-02	10.22
black	BP	Extracellular matrix disassembly	4	2.34	3.49E-02	5.56
black	BP	Calcium ion transport	4	2.34	3.49E-02	5.56
black	BP	Brown fat cell differentiation	3	1.75	3.63E-02	9.90
black	BP	JAK-STAT cascade	3	1.75	3.63E-02	9.90
black	BP	Regulation of vascular endothelial growth factor production	2	1.17	3.71E-02	52.81
black	BP	Negative regulation of smooth muscle contraction	2	1.17	3.71E-02	52.81
black	BP	Neutrophil activation involved in immune response	2	1.17	3.71E-02	52.81
black	BP	T cell costimulation	4	2.34	3.72E-02	5.42
black	BP	Positive regulation of cytosolic calcium ion concentration	5	2.92	3.79E-02	3.94
black	BP	MyD88-dependent toll-like receptor signaling pathway	3	1.75	3.84E-02	9.60
black	BP	Negative regulation of T cell mediated cytotoxicity	2	1.17	4.62E-02	42.24
black	BP	Regulation of platelet activation	2	1.17	4.62E-02	42.24
black	BP	Interleukin-1 beta production	2	1.17	4.62E-02	42.24
black	BP	Proteolysis	10	5.85	4.70E-02	2.11
black	BP	Movement of cell or subcellular component	4	2.34	4.75E-02	4.91
black	BP	Complement activation	4	2.34	4.89E-02	4.86
black	CC	Extracellular region	43	25.15	1.41E-10	2.97
black	CC	Extracellular space	35	20.47	2.73E-08	2.89
black	CC	Integral component of plasma membrane	33	19.30	8.92E-07	2.59
black	CC	Cell surface	18	10.53	7.53E-06	3.69
black	CC	Plasma membrane	62	36.26	1.29E-05	1.67
black	CC	Extracellular exosome	45	26.32	9.95E-05	1.78
black	CC	External side of plasma membrane	10	5.85	1.30E-04	5.22
black	CC	Integral component of membrane	63	36.84	4.50E-03	1.36
black	CC	Early endosome	8	4.68	4.60E-03	3.88
black	CC	Extracellular matrix	9	5.26	5.24E-03	3.38
black	CC	Focal adhesion	10	5.85	8.71E-03	2.84
black	CC	Membrane raft	7	4.09	1.06E-02	3.78

black	CC	Platelet alpha granule lumen	4	2.34	1.31E-02	8.08
black	CC	Lysosome	7	4.09	1.61E-02	3.44
black	CC	Complement component C1 complex	2	1.17	1.78E-02	111.12
black	CC	Actin filament	4	2.34	2.05E-02	6.84
black	CC	Membrane	30	17.54	2.05E-02	1.52
black	CC	Proteinaceous extracellular matrix	7	4.09	3.36E-02	2.90
black	CC	Basement membrane	4	2.34	3.39E-02	5.63
black	CC	NLRP1 inflammasome complex	2	1.17	3.53E-02	55.56
black	CC	Immunological synapse	3	1.75	3.70E-02	9.80
black	CC	Collagen trimer	4	2.34	4.95E-02	4.83
black	MF	Chemokine activity	8	4.68	2.61E-07	18.01
black	MF	Heparin binding	10	5.85	1.46E-05	6.90
black	MF	Transmembrane signaling receptor activity	11	6.43	2.44E-05	5.67
black	MF	Receptor activity	10	5.85	1.56E-04	5.08
black	MF	Protein homodimerization activity	17	9.94	9.09E-04	2.57
black	MF	Cysteine-type endopeptidase activity involved in apoptotic process	3	1.75	5.89E-03	25.46
black	MF	Serine-type endopeptidase activity	8	4.68	8.39E-03	3.46
black	MF	Serine-type endopeptidase inhibitor activity	5	2.92	1.15E-02	5.69
black	MF	Receptor binding	9	5.26	1.47E-02	2.81
black	MF	Integrin binding	5	2.92	1.50E-02	5.25
black	MF	IgE receptor activity	2	1.17	1.79E-02	110.33
black	MF	Cysteine-type endopeptidase inhibitor activity involved in apoptotic process	3	1.75	1.80E-02	14.39
black	MF	CCR chemokine receptor binding	3	1.75	1.80E-02	14.39
black	MF	Cell adhesion molecule binding	4	2.34	1.84E-02	7.12
black	MF	CCR2 chemokine receptor binding	2	1.17	2.68E-02	73.56
black	MF	Actin filament binding	5	2.92	3.15E-02	4.18
black	MF	Carbohydrate binding	6	3.51	3.23E-02	3.38
black	MF	Glycosphingolipid binding	2	1.17	3.55E-02	55.17
black	MF	Deoxycytidine deaminase activity	2	1.17	3.55E-02	55.17
black	MF	IgE binding	2	1.17	4.42E-02	44.13
black	KEGG	Pertussis	9	5.26	3.87E-06	9.42
black	KEGG	Staphylococcus aureus infection	8	4.68	4.31E-06	11.63
black	KEGG	Fc gamma R-mediated phagocytosis	9	5.26	9.12E-06	8.41
black	KEGG	Hematopoietic cell lineage	9	5.26	9.97E-06	8.31
black	KEGG	Chemokine signaling pathway	12	7.02	1.88E-05	5.07
black	KEGG	Cytokine-cytokine receptor interaction	13	7.60	2.70E-05	4.44
black	KEGG	Cell adhesion molecules (CAMs)	10	5.85	6.67E-05	5.53
black	KEGG	Tuberculosis	11	6.43	6.85E-05	4.88
black	KEGG	Phagosome	10	5.85	1.19E-04	5.13
black	KEGG	Malaria	6	3.51	3.48E-04	9.62
black	KEGG	Legionellosis	6	3.51	5.49E-04	8.72
black	KEGG	Leukocyte transendothelial migration	8	4.68	6.70E-04	5.32

black	KEGG	NF-kappa B signaling pathway	7	4.09	7.48E-04	6.32
black	KEGG	Rheumatoid arthritis	7	4.09	7.95E-04	6.25
black	KEGG	Leishmaniasis	6	3.51	1.91E-03	6.64
black	KEGG	TNF signaling pathway	7	4.09	2.09E-03	5.19
black	KEGG	Amoebiasis	7	4.09	2.09E-03	5.19
black	KEGG	Focal adhesion	9	5.26	4.23E-03	3.43
black	KEGG	ECM-receptor interaction	6	3.51	4.64E-03	5.42
black	KEGG	NOD-like receptor signaling pathway	5	2.92	4.90E-03	7.14
black	KEGG	Prion diseases	4	2.34	8.01E-03	9.52
black	KEGG	Primary immunodeficiency	4	2.34	8.71E-03	9.24
black	KEGG	Complement and coagulation cascades	5	2.92	1.09E-02	5.69
black	KEGG	Salmonella infection	5	2.92	2.03E-02	4.73
black	KEGG	Influenza A	7	4.09	2.20E-02	3.16
black	KEGG	Pathogenic Escherichia coli infection	4	2.34	2.60E-02	6.16
black	KEGG	Chagas disease (American trypanosomiasis)	5	2.92	4.17E-02	3.78
purple	BP	Cellular response to nutrient levels	6	14.29	3.30E-05	15.71
purple	BP	Cellular response to extracellular stimulus	6	14.29	7.34E-05	13.28
purple	BP	Cellular response to external stimulus	6	14.29	3.16E-04	9.72
purple	BP	Response to nutrient levels	6	14.29	2.40E-03	6.18
purple	BP	Cellular response to starvation	4	9.52	2.45E-03	14.48
purple	BP	Response to extracellular stimulus	6	14.29	3.29E-03	5.74
purple	BP	Protein maturation	5	11.90	3.61E-03	7.68
purple	BP	Response to acid chemical	5	11.90	3.95E-03	7.49
purple	BP	Response to starvation	4	9.52	5.46E-03	10.88
purple	BP	Renal sodium ion absorption	2	4.76	9.34E-03	208.13
purple	BP	Female pregnancy	4	9.52	9.47E-03	8.90
purple	BP	Response to organic substance	14	33.33	9.58E-03	2.07
purple	BP	Renal sodium ion transport	2	4.76	1.17E-02	166.50
purple	BP	Multi-multicellular organism process	4	9.52	1.38E-02	7.74
purple	BP	Proteolysis	10	23.81	1.45E-02	2.46
purple	BP	Protein processing	4	9.52	1.77E-02	7.06
purple	BP	Proteolysis involved in cellular protein catabolic process	6	14.29	1.79E-02	3.80
purple	BP	Cellular protein catabolic process	6	14.29	2.15E-02	3.62
purple	BP	Pyrimidine-containing compound salvage	2	4.76	2.78E-02	69.38
purple	BP	Pyrimidine nucleoside salvage	2	4.76	2.78E-02	69.38
purple	BP	Protein autoprocessing	2	4.76	3.00E-02	64.04
purple	BP	Cellular catabolic process	9	21.43	3.12E-02	2.32
purple	BP	Homeostatic process	9	21.43	3.42E-02	2.28
purple	BP	Renal absorption	2	4.76	3.46E-02	55.50
purple	BP	Hemoglobin metabolic process	2	4.76	3.46E-02	55.50
purple	BP	Nucleoside biosynthetic process	3	7.14	3.66E-02	9.68
purple	BP	Glycosyl compound biosynthetic process	3	7.14	3.77E-02	9.53

purple	BP	Nucleoside salvage	2	4.76	3.91E-02	48.97
purple	BP	Protein catabolic process	6	14.29	3.96E-02	3.08
purple	BP	Protein stabilization	3	7.14	4.03E-02	9.18
purple	BP	Establishment of mitochondrion localization	2	4.76	4.14E-02	46.25
purple	BP	Positive regulation of protein processing	2	4.76	4.36E-02	43.82
purple	BP	Innate immune response	6	14.29	4.42E-02	2.99
purple	BP	Ubiquitin-dependent protein catabolic process	5	11.90	4.51E-02	3.61
purple	BP	Protein activation cascade	3	7.14	4.58E-02	8.55
purple	BP	Positive regulation of protein maturation	2	4.76	4.58E-02	41.63
purple	BP	Myeloid cell differentiation	4	9.52	4.59E-02	4.85
purple	BP	Response to external stimulus	10	23.81	4.67E-02	2.01
purple	BP	Modification-dependent protein catabolic process	5	11.90	4.70E-02	3.56
purple	BP	Pyrimidine nucleoside catabolic process	2	4.76	4.81E-02	39.64
purple	BP	Inclusion body assembly	2	4.76	4.81E-02	39.64
purple	BP	Response to lipid	6	14.29	4.89E-02	2.90
purple	BP	Modification-dependent macromolecule catabolic process	5	11.90	4.90E-02	3.51
purple	CC	Extracellular space	11	26.19	4.79E-03	2.70
purple	CC	Membrane-bounded vesicle	17	40.48	2.48E-02	1.67
purple	CC	Hemoglobin complex	2	4.76	3.26E-02	59.05
purple	CC	Schmidt-Lanterman incisure	2	4.76	3.26E-02	59.05
purple	CC	Extracellular exosome	14	33.33	3.38E-02	1.76
purple	CC	Extracellular vesicle	14	33.33	3.51E-02	1.76
purple	CC	Extracellular organelle	14	33.33	3.52E-02	1.76
purple	CC	Compact myelin	2	4.76	4.32E-02	44.29
purple	CC	Apicolateral plasma membrane	2	4.76	4.58E-02	41.68
purple	MF	Misfolded protein binding	2	4.76	2.81E-02	68.62
purple	MF	Hydrolase activity	12	28.57	3.57E-02	1.90
purple	MF	Catalytic activity	21	50.00	4.37E-02	1.44

Supplementary Table S5. DEGs identified in mouse diabetic nephropathy

RefSeq Accession	Gene Symbol	GenBank ID	D - C (GSE20636)		GD - GC (GSE33744)	
			logFC	adj.P.Val	logFC	adj.P.Val
NM_001012335	Mdk	17242	-1.05	1.41E-14	-1.28	9.88E-13
NM_001012336	Mdk	17242	-1.05	1.41E-14	-1.28	9.88E-13
NM_001012766	Ear12	503845	0.71	3.72E-04	1.24	2.48E-05
NM_001025610	Ms4a7	109225	0.97	2.43E-06	1.35	4.29E-06
NM_001029937	Sec14l3	380683	-1.58	2.15E-10	-1.60	1.11E-09
NM_001034910	Defb42	619548	0.61	9.33E-05	0.92	9.10E-05
NM_001039150	Cd44	12505	0.84	6.71E-05	1.04	4.71E-06
NM_001039151	Cd44	12505	0.84	6.71E-05	1.04	4.71E-06
NM_001044384	Timp1	21857	0.62	1.51E-04	0.91	3.84E-03

NM_001111048	Fga	14161	0.59	5.89E-04	0.92	1.45E-03
NM_001111099	Cdkn1a	12575	1.02	1.48E-10	1.40	7.89E-16
NM_001145899	Slc15a2	57738	-0.64	1.44E-15	-0.83	2.19E-06
NM_001159986	Sec16b	89867	0.71	8.09E-11	0.83	1.83E-08
NM_001161432	Eda2r	245527	0.64	1.54E-07	1.02	5.90E-10
NM_001164357	Slc25a25	227731	0.60	8.91E-04	0.71	5.19E-06
NM_001164358	Slc25a25	227731	0.60	8.91E-04	0.71	5.19E-06
NM_001166631	Havcr1	171283	0.98	7.29E-05	1.43	1.68E-04
NM_001166632	Havcr1	171283	0.98	7.29E-05	1.43	1.68E-04
NM_001177785	Cd44	12505	0.84	6.71E-05	1.04	4.71E-06
NM_001177786	Cd44	12505	0.84	6.71E-05	1.04	4.71E-06
NM_001177787	Cd44	12505	0.84	6.71E-05	1.04	4.71E-06
NM_001199217	Slc38a3	76257	0.77	1.49E-08	0.87	1.97E-06
NM_001199218	Slc38a3	76257	0.77	1.49E-08	0.87	1.97E-06
NM_001204134	C1qtnf3	81799	-0.97	1.23E-11	-1.68	4.91E-11
NM_001204931	Reep6	70335	1.09	7.47E-10	0.83	5.12E-06
NM_001252330	Slc6a15	103098	-0.89	3.42E-11	-1.18	3.74E-11
NM_001253781	Prlr	19116	-1.13	6.00E-10	0.83	3.66E-06
NM_001276398	Ms4a7	109225	0.88	2.33E-05	1.35	4.96E-06
NM_001281830	Ifi27l2a	76933	0.73	3.59E-03	1.10	1.83E-03
NM_001290273		66112	0.97	1.02E-07	1.52	2.61E-06
NM_001290558	Slc25a25	227731	0.60	8.91E-04	0.71	5.19E-06
NM_001291058	Cd68	12514	0.78	3.06E-06	1.22	1.48E-07
NM_001291481	Mdk	17242	-1.05	1.41E-14	-1.28	9.88E-13
NM_001291482	Mdk	17242	-1.05	1.41E-14	-1.28	9.88E-13
NM_001291483	Mdk	17242	-0.83	1.49E-11	-1.22	5.08E-12
NM_001294280	Timp1	21857	0.59	7.62E-04	0.92	3.42E-03
NM_001311132	Akr1c20	116852	-0.89	3.89E-10	0.69	2.65E-03
NM_001313963	Krt19	16669	0.67	7.78E-05	1.38	1.34E-06
NM_001320076	Mmp12	17381	0.64	6.98E-05	1.30	2.07E-04
NM_001320077	Mmp12	17381	0.64	6.98E-05	1.30	2.07E-04
NM_001347354	#N/A	#N/A	-0.67	6.64E-06	-1.68	7.61E-11
NM_007498	Atf3	11910	0.69	5.19E-05	0.65	3.26E-04
NM_007669	Cdkn1a	12575	1.02	1.48E-10	1.40	7.89E-16
NM_008176	Cxcl1	14825	0.80	1.38E-04	1.20	2.23E-04
NM_008256	Hmgcs2	15360	1.41	5.91E-05	1.74	1.81E-10
NM_008331	Ifit1	15957	0.67	7.96E-05	0.77	2.33E-03
NM_008471	Krt19	16669	0.67	7.78E-05	1.38	1.34E-06
NM_008491	Lcn2	16819	2.03	1.02E-08	3.20	3.53E-09
NM_008586	Mep1b	17288	-0.68	5.41E-09	-1.47	3.83E-06
NM_008605	Mmp12	17381	0.64	6.98E-05	1.30	2.07E-04
NM_008630	Mt2	17750	0.90	8.02E-07	0.65	2.58E-09
NM_008871	Serpine1	18787	0.73	9.44E-05	0.95	1.02E-08
NM_009139	Ccl6	20305	0.63	7.23E-05	1.31	2.13E-08

NM_009264	Sprr1a	20753	0.72	3.60E-04	0.98	4.68E-03
NM_009381	Thrsp	21835	-0.93	5.24E-04	0.76	4.21E-07
NM_009627	Adm	11535	0.69	4.38E-07	0.90	5.13E-10
NM_009778	C3	12266	1.91	1.92E-12	1.40	1.45E-06
NM_009827	Cckar	12425	-0.67	6.64E-06	-1.68	7.61E-11
NM_009851	Cd44	12505	0.84	6.71E-05	1.04	4.71E-06
NM_009899	Clca3a1	12722	0.70	2.05E-06	1.53	5.85E-12
NM_010006	Cyp2d9	13105	0.61	7.57E-04	-0.78	4.74E-03
NM_010493	Icam1	15894	0.60	3.35E-06	0.60	5.95E-07
NM_010594	Kap	16483	-0.83	4.61E-04	-1.32	1.34E-07
NM_010784	Mdk	17242	-1.05	1.41E-14	-1.28	9.88E-13
NM_011076	Abcb1a	18671	-0.60	5.93E-07	0.82	2.83E-07
NM_011169	Prlr	19116	-1.01	5.96E-11	0.76	4.75E-05
NM_011311	S100a4	20198	0.69	5.42E-08	0.82	1.07E-07
NM_011333	Ccl2	20296	0.68	9.82E-06	0.91	4.32E-04
NM_011338	Ccl9	20308	0.70	2.83E-05	1.16	6.60E-08
NM_011414	Slpi	20568	0.73	8.64E-05	2.23	7.83E-11
NM_011593	Timp1	21857	0.62	1.51E-04	0.91	3.84E-03
NM_011671	Ucp2	22228	0.91	1.58E-09	0.94	3.59E-11
NM_011825	Grem2	23893	0.74	2.56E-03	0.60	3.67E-03
NM_013468	Ankrd1	107765	0.97	3.05E-10	1.10	1.91E-09
NM_013602	Mt1	17748	0.62	6.36E-07	0.59	3.19E-10
NM_013697	Ttr	22139	-0.96	2.04E-04	-1.38	1.44E-02
NM_013750	Phlda3	27280	0.61	8.02E-11	0.78	3.98E-10
NM_017372	Lyz2	17105	1.06	2.18E-06	0.94	7.98E-07
NM_017388	Ear3	53876	0.82	4.96E-05	1.46	8.00E-08
NM_018816	Apom	55938	-0.73	3.64E-10	-0.68	1.59E-04
NM_020581	Angptl4	57875	1.15	6.19E-04	1.41	1.16E-09
NM_021274	Cxcl10	15945	0.97	1.58E-06	0.83	1.27E-04
NM_022018	Fam129a	63913	0.61	7.06E-05	1.09	5.69E-10
NM_023256	Krt20	66809	0.79	7.50E-06	1.14	6.49E-05
NM_023805	Slc38a3	76257	0.77	1.49E-08	0.87	1.97E-06
NM_025684	Nepn	66650	-0.70	9.45E-08	-0.74	2.18E-04
NM_027211	Anxa13	69787	-1.55	6.80E-15	-1.75	2.33E-14
NM_027762	Tchhl1	71325	0.72	1.34E-07	0.66	5.69E-03
NM_027836	Ms4a7	109225	0.88	2.33E-05	1.35	4.96E-06
NM_029083	Ddit4	74747	0.67	1.00E-04	0.60	5.70E-06
NM_029662	Mfsd2a	76574	-0.81	6.74E-07	-1.29	1.27E-05
NM_029796	Lrg1	76905	0.69	1.87E-04	1.34	1.94E-09
NM_029803	Ifi27l2a	76933	0.76	4.86E-03	0.99	2.33E-03
NM_030888	C1qtnf3	81799	-0.97	1.23E-11	-1.68	4.91E-11
NM_033354	Sec16b	89867	0.71	8.09E-11	0.83	1.83E-08
NM_053215	Ugt2b37	112417	0.59	1.17E-04	-1.22	2.24E-08
NM_080845	Ftcd	14317	0.62	7.99E-10	0.68	2.02E-03

NM_133888	Smpdl3b	100340	0.95	1.17E-07	1.14	4.89E-08
NM_134248	Havcr1	171283	0.98	7.29E-05	1.43	1.68E-04
NM_138685	Wfdc15b	192201	-0.71	9.78E-12	-0.59	5.31E-05
NM_139292	Reep6	70335	1.09	7.47E-10	0.83	5.12E-06
NM_144785	Slc22a19	207151	-0.59	4.05E-08	-1.77	1.08E-07
NM_144856	Slc22a7	108114	-0.80	1.71E-08	-3.17	3.55E-14
NM_145424	BC089597	216454	-0.60	1.26E-09	0.83	6.71E-05
NM_146118	Slc25a25	227731	0.60	8.91E-04	0.71	5.19E-06
NM_172840	Vwa2	240675	-0.74	2.96E-11	-0.68	6.84E-07
NM_175158	Utp20	70683	0.94	1.11E-07	0.60	3.02E-09
NM_175328	Slc6a15	103098	-0.89	3.42E-11	-1.18	3.74E-11
NM_175540	Eda2r	245527	0.64	1.54E-07	1.02	5.90E-10
NM_177406	Cyp4a12a	277753	1.48	6.14E-10	-1.27	2.97E-09
NM_201360	Cyp2d12	380997	-0.97	6.37E-06	-1.39	7.14E-10
NR_027641	Sec16b	89867	0.71	8.09E-11	0.83	1.83E-08
NR_110993	Cd68	12514	0.78	3.06E-06	1.22	1.48E-07
NR_130895	Clca3a1	12722	0.70	2.05E-06	1.53	5.85E-12
XM_006496826	Grem2	23893	0.74	2.56E-03	0.60	3.67E-03
XM_006497049	Sec16b	89867	0.71	8.09E-11	0.83	1.83E-08
XM_006497954	Slc25a25	227731	0.60	8.91E-04	0.71	5.19E-06
XM_006497955	Slc25a25	227731	0.60	8.91E-04	0.71	5.19E-06
XM_006497957	Slc25a25	227731	0.60	8.91E-04	0.71	5.19E-06
XM_006497958	Slc25a25	227731	0.60	8.91E-04	0.71	5.19E-06
XM_006498645	Cd44	12505	0.84	6.71E-05	1.04	4.71E-06
XM_006498646	Cd44	12505	0.84	6.71E-05	1.04	4.71E-06
XM_006498647	Cd44	12505	0.84	6.71E-05	1.04	4.71E-06
XM_006498649	Cd44	12505	0.84	6.71E-05	1.04	4.71E-06
XM_006501177	S100a4	20198	0.69	5.42E-08	0.82	1.07E-07
XM_006501178	S100a4	20198	0.69	5.42E-08	0.82	1.07E-07
XM_006503477	Mfsd2a	76574	-0.81	6.74E-07	-1.29	1.27E-05
XM_006503555	Abcb1a	18671	-0.60	5.93E-07	0.82	2.83E-07
XM_006503556	Abcb1a	18671	-0.60	5.93E-07	0.82	2.83E-07
XM_006507579	Ucp2	22228	0.91	1.58E-09	0.94	3.59E-11
XM_006512818	Nepn	66650	-0.70	9.45E-08	-0.74	2.18E-04
XM_006512819	Nepn	66650	-0.70	9.45E-08	-0.74	2.18E-04
XM_006512982	Slc6a15	103098	-0.87	6.35E-11	-1.15	2.05E-10
XM_006512983	Slc6a15	103098	-0.87	6.35E-11	-1.15	2.05E-10
XM_006512984	Slc6a15	103098	-0.87	6.35E-11	-1.15	2.05E-10
XM_006513215	Ftcd	14317	0.62	7.99E-10	0.68	2.02E-03
XM_006513216	Ftcd	14317	0.62	7.99E-10	0.68	2.02E-03
XM_006514077	Reep6	70335	1.09	7.47E-10	0.83	5.12E-06
XM_006516465	Akr1c20	116852	-0.89	3.89E-10	0.69	2.65E-03
XM_006516466	Akr1c20	116852	-0.89	3.89E-10	0.69	2.65E-03
XM_006520034	Prlr	19116	-1.01	5.96E-11	0.76	4.75E-05

XM_006520035	Prlr	19116	-1.01	5.96E-11	0.76	4.75E-05
XM_006520036	Prlr	19116	-1.01	5.96E-11	0.76	4.75E-05
XM_006520037	Prlr	19116	-1.01	5.96E-11	0.76	4.75E-05
XM_006521128	Cyp2d12	380997	-0.97	6.37E-06	-1.39	7.14E-10
XM_006521129	Cyp2d12	380997	-0.97	6.37E-06	-1.39	7.14E-10
XM_006521130	Cyp2d12	380997	-0.97	6.37E-06	-1.39	7.14E-10
XM_006521131	Cyp2d12	380997	-0.97	6.37E-06	-1.39	7.14E-10
XM_006521132	Cyp2d12	380997	-0.97	6.37E-06	-1.39	7.14E-10
XM_006526814	Slc22a19	207151	-0.59	4.05E-08	-1.77	1.08E-07
XM_006527090	Vwa2	240675	-0.74	2.96E-11	-0.68	6.84E-07
XM_006527091	Vwa2	240675	-0.74	2.96E-11	-0.68	6.84E-07
XM_006528025	Eda2r	245527	0.64	1.54E-07	1.02	5.90E-10
XM_006528026	Eda2r	245527	0.64	1.54E-07	1.02	5.90E-10
XM_006530752	Mt1	17748	0.62	4.45E-07	0.76	6.32E-11
XM_006532392	Havcr1	171283	0.97	7.65E-05	1.45	2.03E-04
XM_006532393	Havcr1	171283	0.98	7.29E-05	1.43	1.68E-04
XM_011239335	Mdk	17242	-1.05	1.41E-14	-1.28	9.88E-13
XM_011240004	Clca3a1	12722	0.70	2.05E-06	1.53	5.85E-12
XM_011243730	Sec14l3	380683	-1.52	2.71E-10	-1.60	1.62E-09
XM_011246258	C3	12266	1.91	1.92E-12	1.40	1.45E-06
XM_011248783	Havcr1	171283	0.97	7.65E-05	1.45	2.03E-04
XM_011248784	Havcr1	171283	0.97	7.65E-05	1.45	2.03E-04
XM_017312983	Sec16b	89867	0.71	8.09E-11	0.83	1.83E-08
XM_017312984	Sec16b	89867	0.71	8.09E-11	0.83	1.83E-08
XM_017313160	Mmp12	17381	0.64	6.98E-05	1.30	2.07E-04
XM_017313682	Slc38a3	76257	0.77	1.49E-08	0.87	1.97E-06
XM_017318188	Slc22a26	236149	-1.26	6.41E-07	-0.97	3.33E-06
XM_017320448	Grem2	23893	0.74	2.56E-03	0.60	3.67E-03
XR_383836	C1qtnf3	81799	-0.97	1.23E-11	-1.68	4.91E-11
XR_871103	Slc38a3	76257	0.77	1.49E-08	0.87	1.97E-06
XR_875302	Cyp2d12	380997	-0.97	6.37E-06	-1.39	7.14E-10

Supplementary Table S6. Functional enrichment of DEGs identified in human diabetic nephropathy

Category	GO Term/Pathway	Gene count	Gene Ratio (%)	P Value	Fold Enrichment
BP	Defense response	49	38.28	3.04E-20	4.52
BP	Immune response	47	36.72	3.24E-18	4.24
BP	Immune system process	57	44.53	2.75E-17	3.24
BP	Innate immune response	32	25.00	1.31E-14	5.36
BP	Regulation of immune system process	39	30.47	8.77E-14	3.96
BP	Cell activation	32	25.00	1.29E-13	4.92

BP	Regulation of immune response	32	25.00	1.37E-13	4.91
BP	Response to stress	63	49.22	4.52E-13	2.40
BP	Positive regulation of immune system process	32	25.00	5.32E-13	4.66
BP	Immune effector process	28	21.88	1.45E-12	5.28
BP	Leukocyte activation	28	21.88	1.70E-12	5.25
BP	Positive regulation of immune response	26	20.31	1.03E-11	5.32
BP	Response to external stimulus	44	34.38	1.99E-11	2.97
BP	Inflammatory response	24	18.75	9.97E-11	5.27
BP	Adaptive immune response	20	15.63	1.21E-10	6.66
BP	Regulation of response to external stimulus	25	19.53	1.94E-10	4.85
BP	Leukocyte cell-cell adhesion	21	16.41	2.40E-10	5.98
BP	Humoral immune response	16	12.50	2.40E-10	9.03
BP	Biological adhesion	38	29.69	2.49E-10	3.12
BP	Activation of immune response	22	17.19	2.64E-10	5.60
BP	Positive regulation of response to stimulus	41	32.03	6.47E-10	2.83
BP	Cell adhesion	37	28.91	9.16E-10	3.05
BP	T cell aggregation	19	14.84	2.41E-09	5.96
BP	T cell activation	19	14.84	2.41E-09	5.96
BP	Lymphocyte aggregation	19	14.84	2.50E-09	5.95
BP	Response to other organism	25	19.53	3.16E-09	4.22
BP	Response to external biotic stimulus	25	19.53	3.16E-09	4.22
BP	Leukocyte aggregation	19	14.84	3.19E-09	5.86
BP	Lymphocyte activation	22	17.19	4.35E-09	4.79
BP	Single organism cell adhesion	24	18.75	4.91E-09	4.31
BP	Response to stimulus	91	71.09	5.03E-09	1.53
BP	Single organismal cell-cell adhesion	23	17.97	6.80E-09	4.44
BP	Myeloid leukocyte activation	12	9.38	7.46E-09	11.42
BP	Response to biotic stimulus	25	19.53	8.66E-09	4.01
BP	Leukocyte migration	17	13.28	9.60E-09	6.33
BP	Regulation of response to stimulus	54	42.19	1.93E-08	2.08

BP	Leukocyte mediated immunity	16	12.50	3.29E-08	6.29
BP	Positive regulation of leukocyte activation	15	11.72	4.30E-08	6.75
BP	Positive regulation of biological process	67	52.34	4.86E-08	1.77
BP	Acute inflammatory response	11	8.59	5.03E-08	11.15
BP	Positive regulation of cell activation	15	11.72	6.13E-08	6.56
BP	Regulation of response to stress	29	22.66	6.25E-08	3.16
BP	Response to organic substance	45	35.16	9.46E-08	2.23
BP	Lymphocyte mediated immunity	14	10.94	1.16E-07	6.87
BP	Adaptive immune response based on somatic recombination of immune receptors built from immunoglobulin superfamily domains	14	10.94	1.21E-07	6.85
BP	Complement activation	10	7.81	1.44E-07	11.96
BP	Regulation of defense response	20	15.63	2.72E-07	4.14
BP	Regulation of inflammatory response	14	10.94	3.53E-07	6.24
BP	Response to molecule of bacterial origin	14	10.94	3.79E-07	6.20
BP	Cell-cell adhesion	26	20.31	4.86E-07	3.13
BP	Regulation of cell activation	17	13.28	4.93E-07	4.75
BP	Regulation of immune effector process	14	10.94	5.21E-07	6.03
BP	Positive regulation of multicellular organismal process	29	22.66	5.77E-07	2.84
BP	Regulation of cytokine production	18	14.06	6.47E-07	4.37
BP	Positive regulation of cellular process	60	46.88	6.53E-07	1.77
BP	Regulation of acute inflammatory response	8	6.25	9.13E-07	15.13
BP	Positive regulation of lymphocyte activation	13	10.16	9.21E-07	6.34
BP	Protein activation cascade	10	7.81	9.52E-07	9.58
BP	Regulation of leukocyte activation	16	12.50	1.11E-06	4.78
BP	Cellular response to chemical stimulus	41	32.03	1.13E-06	2.17
BP	Regulation of lymphocyte activation	15	11.72	1.27E-06	5.11
BP	Humoral immune response mediated by circulating immunoglobulin	9	7.03	1.29E-06	11.24
BP	Regulation of T cell activation	13	10.16	1.42E-06	6.08
BP	Cytokine production	18	14.06	2.20E-06	3.99
BP	Regulation of leukocyte cell-cell adhesion	13	10.16	2.28E-06	5.81

BP	Cell migration	25	19.53	2.39E-06	2.95
BP	Positive regulation of T cell activation	11	8.59	2.46E-06	7.33
BP	Regulation of cell adhesion	18	14.06	2.83E-06	3.92
BP	Regulation of biological process	101	78.91	3.08E-06	1.29
BP	Positive regulation of leukocyte cell-cell adhesion	11	8.59	3.43E-06	7.06
BP	Immunoglobulin mediated immune response	10	7.81	3.54E-06	8.18
BP	Regulation of complement activation	6	4.69	3.88E-06	24.69
BP	B cell mediated immunity	10	7.81	3.89E-06	8.09
BP	Leukocyte proliferation	12	9.38	4.88E-06	6.00
BP	Regulation of protein activation cascade	6	4.69	5.19E-06	23.32
BP	Cellular response to organic substance	35	27.34	5.31E-06	2.24
BP	Complement activation, classical pathway	8	6.25	6.53E-06	11.31
BP	Response to bacterium	16	12.50	6.96E-06	4.12
BP	Regulation of phagocytosis	7	5.47	7.83E-06	14.62
BP	Response to chemical	52	40.63	8.27E-06	1.77
BP	Cytokine-mediated signaling pathway	16	12.50	8.66E-06	4.04
BP	Regulation of multicellular organismal process	39	30.47	9.11E-06	2.05
BP	Regulation of tumor necrosis factor production	8	6.25	9.64E-06	10.66
BP	Cellular response to cytokine stimulus	18	14.06	9.77E-06	3.57
BP	Leukocyte chemotaxis	10	7.81	9.86E-06	7.21
BP	Regulation of proteolysis	18	14.06	1.01E-05	3.56
BP	Positive regulation of cell-cell adhesion	11	8.59	1.07E-05	6.21
BP	Cell proliferation	31	24.22	1.13E-05	2.33
BP	Regulation of tumor necrosis factor superfamily cytokine production	8	6.25	1.16E-05	10.37
BP	Cell chemotaxis	11	8.59	1.19E-05	6.13
BP	Response to cytokine	19	14.84	1.36E-05	3.32
BP	Tumor necrosis factor superfamily cytokine production	8	6.25	1.39E-05	10.09
BP	Positive regulation of phagocytosis	6	4.69	1.43E-05	19.08
BP	Proteolysis	29	22.66	1.51E-05	2.40
BP	Positive regulation of endocytosis	8	6.25	1.56E-05	9.91

BP	Biological regulation	103	80.47	1.58E-05	
CC	Extracellular region	71	55.47	5.35E-14	2.275480146
CC	Extracellular space	38	29.69	3.89E-13	3.899015832
CC	Extracellular region part	63	49.22	5.14E-13	2.406973673
CC	Membrane-bounded vesicle	57	44.53	5.98E-11	2.338761643
CC	Extracellular exosome	49	38.28	1.24E-10	2.58269921
CC	Extracellular vesicle	49	38.28	1.47E-10	2.569899993
CC	Extracellular organelle	49	38.28	1.49E-10	2.568990616
CC	Vesicle	57	44.53	2.85E-10	2.249671895
CC	Blood microparticle	11	8.59	8.49E-08	10.58304297
CC	Extracellular matrix component	10	7.81	1.96E-07	11.57520325
CC	Proteinaceous extracellular matrix	13	10.16	5.59E-06	5.335495349
CC	Extracellular matrix	15	11.72	1.25E-05	4.193281178
CC	Cell periphery	58	45.31	2.05E-05	1.634975437
CC	Basement membrane	7	5.47	4.25E-05	10.91724433
CC	Plasma membrane	55	42.97	1.06E-04	1.585089105
CC	Integral component of plasma membrane	25	19.53	1.89E-04	2.265483205
CC	Plasma membrane part	34	26.56	2.03E-04	1.911775505
CC	Intrinsic component of plasma membrane	25	19.53	3.41E-04	2.177580859
CC	Collagen trimer	6	4.69	6.15E-04	8.715447154
MF	Peptidase regulator activity	14	10.94	3.32E-09	9.260736677
MF	Serine-type endopeptidase activity	13	10.16	1.70E-07	7.418965517
MF	Serine-type peptidase activity	13	10.16	4.99E-07	6.708639032
MF	Serine hydrolase activity	13	10.16	5.59E-07	6.638021779
MF	Endopeptidase activity	16	12.50	1.34E-06	4.703866249
MF	Endopeptidase inhibitor activity	10	7.81	2.94E-06	8.363555291
MF	Endopeptidase regulator activity	10	7.81	3.89E-06	8.084770115
MF	Peptidase inhibitor activity	10	7.81	4.45E-06	7.9522329
MF	Enzyme regulator activity	21	16.41	1.19E-05	3.068316369
MF	Glycosaminoglycan binding	10	7.81	1.25E-05	6.996435676

MF	Molecular function regulator	25	19.53	1.95E-05	2.604256658
MF	Peptidase activity, acting on L-amino acid peptides	17	13.28	2.41E-05	3.49426505
MF	Peptidase activity	17	13.28	3.67E-05	3.375088206
MF	Receptor binding	25	19.53	4.08E-05	2.488472334
MF	Complement binding	4	3.13	3.23E-04	29.10517241
MF	Enzyme inhibitor activity	11	8.59	3.27E-04	4.115127205
MF	Transmembrane signaling receptor activity	21	16.41	5.07E-04	2.334639498
MF	Heparin binding	7	5.47	7.93E-04	6.366756466
MF	Transmembrane receptor activity	21	16.41	8.43E-04	2.24214461
MF	Molecular transducer activity	24	18.75	8.57E-04	2.078940887
MF	Receptor activity	24	18.75	8.57E-04	2.078940887
MF	Signaling receptor activity	21	16.41	1.36E-03	2.156699438
MF	Identical protein binding	20	15.63	2.00E-03	2.143238028
MF	Protein complex binding	14	10.94	2.14E-03	2.659741604
MF	Peptidase activator activity	4	3.13	2.18E-03	15.3185118
MF	Extracellular matrix structural constituent	5	3.91	2.38E-03	8.873528175
MF	Cysteine-type endopeptidase regulator activity involved in apoptotic process	4	3.13	2.72E-03	14.19764508
MF	Protein homodimerization activity	13	10.16	4.10E-03	2.591556448
MF	Sulfur compound binding	7	5.47	5.23E-03	4.372021607
MF	Protein dimerization activity	17	13.28	5.32E-03	2.132706599
MF	Cysteine-type endopeptidase activator activity involved in apoptotic process	3	2.34	5.85E-03	25.68103448
MF	Signal transducer activity	22	17.19	7.67E-03	1.815977859
MF	Peptidase activator activity involved in apoptotic process	3	2.34	8.07E-03	21.82887931
MF	Chemokine receptor binding	4	3.13	9.09E-03	9.239737274
MF	Carbohydrate binding	7	5.47	9.42E-03	3.858640282
MF	Collagen binding	4	3.13	9.49E-03	9.095366379
MF	Immunoglobulin binding	3	2.34	9.72E-03	19.84443574
MF	Hydrolase activity	28	21.88	1.49E-02	1.565395366
MF	Antigen binding	5	3.91	1.94E-02	4.850862069
MF	Enzyme activator activity	9	7.03	1.97E-02	2.651280888

MF	Cysteine-type endopeptidase activity	4	3.13	2.16E-02	6.690844233
MF	Cytokine receptor activity	4	3.13	2.36E-02	6.467816092
MF	Protein binding	85	66.41	2.52E-02	1.144706485
MF	Deoxycytidine deaminase activity	2	1.56	2.70E-02	72.76293103
MF	Serine-type endopeptidase inhibitor activity	4	3.13	2.86E-02	6.001066477
MF	CCR chemokine receptor binding	3	2.34	3.03E-02	10.91443966
MF	G-protein coupled receptor binding	6	4.69	3.13E-02	3.397490943
MF	Macromolecular complex binding	16	12.50	3.17E-02	1.782858953
MF	Cell adhesion molecule binding	8	6.25	3.83E-02	2.52539457
MF	Cytokine receptor binding	6	4.69	3.90E-02	3.198370595
MF	Chemokine activity	3	2.34	4.40E-02	8.909746657
MF	Protease binding	4	3.13	4.58E-02	4.975243148
MF	Retinal dehydrogenase activity	2	1.56	4.67E-02	41.57881773
KEGG	Pertussis	10	7.81	3.77E-08	13.352657
KEGG	Staphylococcus aureus infection	8	6.25	8.01E-07	14.83628556
KEGG	Complement and coagulation cascades	7	5.47	5.35E-05	10.15963033
KEGG	Focal adhesion	10	7.81	1.67E-04	4.861404249
KEGG	Systemic lupus erythematosus	8	6.25	3.19E-04	5.978801644
KEGG	Phagosome	8	6.25	7.11E-04	5.23633608
KEGG	ECM-receptor interaction	6	4.69	1.58E-03	6.906546727
KEGG	Tuberculosis	8	6.25	1.67E-03	4.526324408
KEGG	Prion diseases	4	3.13	4.03E-03	12.1387791
KEGG	Cytokine-cytokine receptor interaction	8	6.25	7.13E-03	3.483301827
KEGG	Chemokine signaling pathway	7	5.47	9.64E-03	3.768895122
KEGG	Rheumatoid arthritis	5	3.91	1.08E-02	5.690052701
KEGG	Cell adhesion molecules (CAMs)	6	4.69	1.26E-02	4.231475811
KEGG	Legionellosis	4	3.13	1.58E-02	7.41814278
KEGG	Regulation of actin cytoskeleton	7	5.47	1.71E-02	3.322343568
KEGG	Chagas disease (American trypanosomiasis)	5	3.91	1.89E-02	4.814659978
KEGG	PI3K-Akt signaling pathway	9	7.03	1.92E-02	2.612476371

KEGG	Amoebiasis	5	3.91	2.01E-02	4.723817337
KEGG	Influenza A	6	4.69	2.78E-02	3.453273363
KEGG	Leukocyte transendothelial migration	5	3.91	2.85E-02	4.243429133
KEGG	Natural killer cell mediated cytotoxicity	5	3.91	3.17E-02	4.104300309
KEGG	Primary immunodeficiency	3	2.34	4.37E-02	8.836317136
KEGG	Fc gamma R-mediated phagocytosis	4	3.13	4.94E-02	4.768806073

Supplementary Table S7. Functional enrichment of DEGs identified in mouse diabetic nephropathy

Category	GO Term/Pathway	Gene count	Gene Ratio (%)	P Value	Fold Enrichment
BP	Response to external stimulus	30	37.97	2.78E-09	3.27
BP	Response to stress	37	46.84	3.43E-09	2.64
BP	Defense response	22	27.85	1.33E-07	3.73
BP	Cellular response to cytokine stimulus	14	17.72	1.62E-07	6.49
BP	Response to cytokine	15	18.99	2.94E-07	5.59
BP	Response to nutrient levels	12	15.19	8.59E-07	7.01
BP	Response to extracellular stimulus	12	15.19	1.73E-06	6.52
BP	Response to other organism	17	21.52	1.74E-06	4.16
BP	Response to external biotic stimulus	17	21.52	1.76E-06	4.15
BP	Cellular response to interleukin-1	7	8.86	2.07E-06	18.31
BP	Response to organic substance	29	36.71	3.00E-06	2.45
BP	Response to biotic stimulus	17	21.52	3.50E-06	3.94
BP	Response to chemical	34	43.04	7.94E-06	2.08
BP	Response to interleukin-1	7	8.86	7.96E-06	14.53
BP	ERK1 and ERK2 cascade	9	11.39	8.92E-06	8.56
BP	Positive regulation of ERK1 and ERK2 cascade	8	10.13	9.54E-06	10.57
BP	Response to bacterium	13	16.46	1.08E-05	4.86
BP	Regulation of ERK1 and ERK2 cascade	9	11.39	1.17E-05	8.25
BP	Inflammatory response	12	15.19	2.51E-05	4.92
BP	Single-organism transport	28	35.44	2.60E-05	2.25
BP	Response to starvation	7	8.86	4.47E-05	10.72

BP	Cellular response to nutrient levels	7	8.86	5.46E-05	10.34
BP	Localization	39	49.37	6.99E-05	1.74
BP	Monocyte chemotaxis	5	6.33	7.37E-05	22.03
BP	Single-organism localization	28	35.44	7.87E-05	2.11
BP	Regulation of cell communication	25	31.65	9.39E-05	2.25
BP	Chemokine-mediated signaling pathway	5	6.33	1.03E-04	20.25
BP	Response to stimulus	52	65.82	1.03E-04	1.46
BP	Cellular response to external stimulus	8	10.13	1.03E-04	7.28
BP	Regulation of signaling	25	31.65	1.07E-04	2.24
BP	Signal transduction by p53 class mediator	6	7.59	1.09E-04	12.56
BP	Cellular response to organic substance	21	26.58	1.18E-04	2.51
BP	Regulation of signal transduction	23	29.11	1.26E-04	2.34
BP	Regulation of inflammatory response	8	10.13	1.29E-04	7.02
BP	Apoptotic process	19	24.05	1.31E-04	2.68
BP	Cellular response to extracellular stimulus	7	8.86	1.38E-04	8.75
BP	Defense response to other organism	11	13.92	1.39E-04	4.48
BP	Aging	8	10.13	1.63E-04	6.76
BP	Response to lipopolysaccharide	9	11.39	1.68E-04	5.65
BP	Negative regulation of endothelial cell apoptotic process	4	5.06	1.77E-04	35.88
BP	Regulation of response to external stimulus	12	15.19	1.91E-04	3.92
BP	Cell death	20	25.32	1.91E-04	2.51
BP	Transport	32	40.51	1.95E-04	1.86
BP	Positive regulation of phosphate metabolic process	14	17.72	2.04E-04	3.34
BP	Positive regulation of phosphorus metabolic process	14	17.72	2.04E-04	3.34
BP	Intrinsic apoptotic signaling pathway by p53 class mediator	5	6.33	2.04E-04	16.97
BP	Response to molecule of bacterial origin	9	11.39	2.13E-04	5.46
BP	Regulation of response to stimulus	27	34.18	2.19E-04	2.04
BP	Programmed cell death	19	24.05	2.46E-04	2.55
BP	Cytokine-mediated signaling pathway	8	10.13	2.87E-04	6.16
BP	Cellular response to tumor necrosis factor	6	7.59	2.99E-04	10.11

BP	Response to drug	9	11.39	3.57E-04	5.06
BP	Establishment of localization	32	40.51	3.61E-04	1.80
BP	Apoptotic signaling pathway	10	12.66	4.39E-04	4.32
BP	Response to lipid	13	16.46	4.40E-04	3.29
BP	Regulation of cell death	17	21.52	4.44E-04	2.64
BP	Myeloid leukocyte migration	6	7.59	4.51E-04	9.24
BP	Positive regulation of MAPK cascade	9	11.39	4.77E-04	4.84
BP	Response to tumor necrosis factor	6	7.59	5.04E-04	9.02
BP	Intrinsic apoptotic signaling pathway in response to DNA damage by p53 class mediator	4	5.06	5.16E-04	25.11
BP	Regulation of defense response	10	12.66	5.67E-04	4.16
BP	Positive regulation of response to stimulus	18	22.78	5.92E-04	2.46
BP	Response to oxygen levels	8	10.13	6.23E-04	5.42
BP	Positive regulation of response to external stimulus	7	8.86	6.66E-04	6.51
BP	Positive regulation of protein phosphorylation	12	15.19	6.78E-04	3.37
BP	Regulation of endothelial cell apoptotic process	4	5.06	6.85E-04	22.83
BP	Ion transport	15	18.99	6.88E-04	2.78
BP	Cell migration	14	17.72	7.08E-04	2.93
BP	Negative regulation of epithelial cell apoptotic process	4	5.06	7.31E-04	22.32
BP	Cellular chemical homeostasis	11	13.92	7.50E-04	3.63
BP	Positive regulation of intracellular signal transduction	12	15.19	7.57E-04	3.33
BP	Cellular response to chemical stimulus	22	27.85	7.65E-04	2.12
BP	Positive regulation of defense response	7	8.86	7.91E-04	6.30
BP	Positive regulation of immune system process	11	13.92	9.15E-04	3.53
BP	Immune response	14	17.72	9.18E-04	2.85
BP	Single-organism metabolic process	27	34.18	9.35E-04	1.86
BP	Regulation of extrinsic apoptotic signaling pathway via death domain receptors	4	5.06	9.39E-04	20.50
BP	Endothelial cell apoptotic process	4	5.06	9.39E-04	20.50
BP	Response to wounding	9	11.39	9.50E-04	4.36
BP	Positive regulation of phosphorylation	12	15.19	9.81E-04	3.23

BP	Positive regulation of inflammatory response	5	6.33	9.85E-04	11.21
BP	Leukocyte chemotaxis	6	7.59	1.04E-03	7.69
BP	Response to hypoxia	7	8.86	1.13E-03	5.88
BP	Defense response to bacterium	7	8.86	1.17E-03	5.84
BP	Leukocyte migration	7	8.86	1.21E-03	5.80
BP	Response to endogenous stimulus	16	20.25	1.24E-03	2.50
BP	Regulation of MAPK cascade	10	12.66	1.26E-03	3.72
BP	Response to decreased oxygen levels	7	8.86	1.32E-03	5.71
BP	Positive regulation of monocyte chemotaxis	3	3.80	1.34E-03	53.82
BP	Regulation of phosphate metabolic process	16	20.25	1.45E-03	2.46
BP	Regulation of phosphorus metabolic process	16	20.25	1.45E-03	2.46
BP	MAPK cascade	10	12.66	1.48E-03	3.63
BP	Cellular response to starvation	5	6.33	1.48E-03	10.05
BP	Signal transduction by protein phosphorylation	10	12.66	1.54E-03	3.61
BP	Regulation of protein phosphorylation	14	17.72	1.58E-03	2.69
BP	Intracellular signal transduction	20	25.32	1.64E-03	2.11
BP	Regulation of apoptotic process	15	18.99	1.65E-03	2.54
BP	Positive regulation of biological process	34	43.04	1.67E-03	1.61
BP	Lymphocyte chemotaxis	4	5.06	1.69E-03	16.74
BP	Positive regulation of leukocyte migration	5	6.33	1.71E-03	9.66
BP	Positive regulation of cell communication	15	18.99	1.74E-03	2.53
BP	Negative regulation of cell communication	13	16.46	1.74E-03	2.81
BP	Multi-organism process	20	25.32	1.75E-03	2.10
BP	Negative regulation of signaling	13	16.46	1.79E-03	2.80
BP	Positive regulation of signal transduction	14	17.72	1.80E-03	2.65
BP	Positive regulation of signaling	15	18.99	1.81E-03	2.52
BP	Response to oxygen-containing compound	16	20.25	1.82E-03	2.41
BP	Regulation of programmed cell death	15	18.99	1.82E-03	2.51
BP	Cellular homeostasis	11	13.92	1.96E-03	3.19
BP	Cellular response to stress	16	20.25	2.04E-03	2.38

BP	Cell motility	14	17.72	2.06E-03	2.61
BP	Localization of cell	14	17.72	2.06E-03	2.61
BP	Negative regulation of signal transduction	12	15.19	2.06E-03	2.94
BP	Negative regulation of response to stimulus	14	17.72	2.09E-03	2.60
BP	Regulation of response to stress	13	16.46	2.17E-03	2.74
BP	Regulation of angiogenesis	6	7.59	2.26E-03	6.44
BP	Negative regulation of cell death	12	15.19	2.35E-03	2.89
BP	Cellular response to lipopolysaccharide	6	7.59	2.39E-03	6.36
BP	Regulation of intracellular signal transduction	15	18.99	2.54E-03	2.43
BP	Organic substance transport	20	25.32	2.61E-03	2.03
BP	Hormone transport	7	8.86	2.73E-03	4.94
BP	Cellular response to molecule of bacterial origin	6	7.59	2.76E-03	6.15
BP	Regulation of monocyte chemotaxis	3	3.80	2.76E-03	37.67
BP	Regulation of phosphorylation	14	17.72	3.00E-03	2.50
BP	Positive regulation of protein modification process	12	15.19	3.14E-03	2.79
BP	Regulation of epithelial cell apoptotic process	4	5.06	3.32E-03	13.22
BP	Extrinsic apoptotic signaling pathway via death domain receptors	4	5.06	3.32E-03	13.22
BP	Immune system process	19	24.05	3.42E-03	2.04
BP	Regulation of vasculature development	6	7.59	3.44E-03	5.84
BP	Negative regulation of apoptotic process	11	13.92	3.45E-03	2.95
BP	Response to temperature stimulus	5	6.33	3.54E-03	7.90
BP	Chemical homeostasis	12	15.19	3.59E-03	2.74
BP	Cell chemotaxis	6	7.59	3.62E-03	5.77
BP	Innate immune response	9	11.39	3.62E-03	3.52
BP	Cellular response to biotic stimulus	6	7.59	3.86E-03	5.69
BP	Negative regulation of programmed cell death	11	13.92	3.86E-03	2.90
BP	Response to glucocorticoid	5	6.33	3.95E-03	7.66
BP	Cellular metal ion homeostasis	8	10.13	4.16E-03	3.88
BP	Organ regeneration	4	5.06	4.26E-03	12.10
BP	Response to copper ion	3	3.80	4.30E-03	30.14

BP	Regulation of transport	16	20.25	4.35E-03	2.20
BP	Lymphocyte migration	4	5.06	4.40E-03	11.96
BP	Negative regulation of response to external stimulus	6	7.59	4.51E-03	5.48
BP	Positive regulation of metabolic process	22	27.85	4.59E-03	1.84
BP	Negative regulation of extrinsic apoptotic signaling pathway via death domain receptors	3	3.80	4.65E-03	28.98
BP	Regulation of leukocyte migration	5	6.33	4.68E-03	7.30
BP	Response to hormone	10	12.66	4.73E-03	3.06
BP	Neutrophil chemotaxis	4	5.06	4.86E-03	11.55
BP	Cellular response to amino acid starvation	3	3.80	5.01E-03	27.90
BP	Positive regulation of leukocyte chemotaxis	4	5.06	5.01E-03	11.42
BP	Wound healing	7	8.86	5.18E-03	4.33
BP	Response to corticosteroid	5	6.33	5.60E-03	6.94
BP	Positive regulation of transport	11	13.92	5.68E-03	2.75
BP	Locomotion	14	17.72	6.07E-03	2.30
BP	Organic anion transport	6	7.59	6.14E-03	5.09
BP	Positive regulation of cellular process	30	37.97	6.24E-03	1.57
BP	Anion transport	7	8.86	6.30E-03	4.16
BP	Cellular response to endogenous stimulus	12	15.19	6.38E-03	2.53
BP	Response to heat	4	5.06	6.76E-03	10.25
BP	Cellular divalent inorganic cation homeostasis	7	8.86	7.11E-03	4.05
BP	Epithelial cell apoptotic process	4	5.06	7.34E-03	9.95
BP	Neutrophil migration	4	5.06	7.34E-03	9.95
BP	Angiogenesis	7	8.86	7.91E-03	3.96
BP	Cellular cation homeostasis	8	10.13	8.10E-03	3.42
BP	Cellular response to transforming growth factor beta stimulus	5	6.33	8.20E-03	6.22
BP	Small molecule metabolic process	15	18.99	8.25E-03	2.13
BP	Granulocyte chemotaxis	4	5.06	8.39E-03	9.48
BP	Metal ion homeostasis	8	10.13	8.39E-03	3.40
BP	Intrinsic apoptotic signaling pathway in response to DNA damage	4	5.06	8.60E-03	9.39
BP	Response to transforming growth factor beta	5	6.33	8.63E-03	6.13

BP	Female pregnancy	5	6.33	8.92E-03	6.07
BP	Cellular response to growth factor stimulus	8	10.13	9.00E-03	3.35
BP	Regulation of leukocyte chemotaxis	4	5.06	9.05E-03	9.22
BP	Regulation of immune system process	12	15.19	9.11E-03	2.41
BP	Divalent inorganic cation homeostasis	7	8.86	9.14E-03	3.84
BP	Cellular ion homeostasis	8	10.13	9.24E-03	3.34
BP	Regulation of protein modification process	14	17.72	9.61E-03	2.17
BP	Positive regulation of cell death	8	10.13	1.08E-02	3.24
BP	Response to growth factor	8	10.13	1.10E-02	3.22
BP	Positive regulation of cell proliferation	10	12.66	1.12E-02	2.66
BP	Granulocyte migration	4	5.06	1.15E-02	8.44
BP	Metal ion transport	9	11.39	1.17E-02	2.88
BP	Antibacterial humoral response	3	3.80	1.18E-02	17.94
BP	Interspecies interaction between organisms	8	10.13	1.25E-02	3.14
BP	Symbiosis, encompassing mutualism through parasitism	8	10.13	1.25E-02	3.14
BP	Regulation of calcium ion transport	5	6.33	1.29E-02	5.44
BP	Multi-multicellular organism process	5	6.33	1.33E-02	5.39
BP	Extrinsic apoptotic signaling pathway	5	6.33	1.35E-02	5.37
BP	Response to abiotic stimulus	11	13.92	1.38E-02	2.41
BP	Regulation of metal ion transport	6	7.59	1.39E-02	4.16
BP	Cell surface receptor signaling pathway	17	21.52	1.39E-02	1.87
BP	Antimicrobial humoral response	3	3.80	1.41E-02	16.38
BP	Negative regulation of endopeptidase activity	4	5.06	1.43E-02	7.79
BP	Positive regulation of cellular protein metabolic process	12	15.19	1.44E-02	2.26
BP	Regulation of hormone levels	7	8.86	1.48E-02	3.45
BP	Regulation of cellular protein metabolic process	17	21.52	1.50E-02	1.86
BP	Positive regulation of chemotaxis	4	5.06	1.52E-02	7.61
BP	Detoxification of copper ion	2	2.53	1.56E-02	125.57
BP	Stress response to copper ion	2	2.53	1.56E-02	125.57
BP	Movement of cell or subcellular component	14	17.72	1.58E-02	2.04

BP	Cation homeostasis	8	10.13	1.58E-02	3.00
BP	Positive regulation of angiogenesis	4	5.06	1.61E-02	7.44
BP	Response to organic cyclic compound	10	12.66	1.65E-02	2.50
BP	Protein phosphorylation	14	17.72	1.67E-02	2.02
BP	Positive regulation of ion transport	5	6.33	1.68E-02	5.02
BP	Inorganic ion homeostasis	8	10.13	1.78E-02	2.93
BP	Blood vessel morphogenesis	7	8.86	1.92E-02	3.26
BP	Positive regulation of apoptotic cell clearance	2	2.53	1.95E-02	100.46
BP	Response to toxic substance	4	5.06	1.98E-02	6.88
BP	DNA damage response, signal transduction by p53 class mediator	3	3.80	2.04E-02	13.45
BP	Response to gamma radiation	3	3.80	2.04E-02	13.45
BP	Monocarboxylic acid metabolic process	7	8.86	2.13E-02	3.18
BP	Response to ionizing radiation	4	5.06	2.16E-02	6.65
BP	Positive regulation of vasculature development	4	5.06	2.20E-02	6.61
BP	Defense response to Gram-negative bacterium	3	3.80	2.25E-02	12.77
BP	Cellular response to oxygen-containing compound	10	12.66	2.28E-02	2.36
BP	Positive regulation of protein metabolic process	12	15.19	2.28E-02	2.11
BP	Ion homeostasis	8	10.13	2.31E-02	2.78
BP	Intrinsic apoptotic signaling pathway	5	6.33	2.32E-02	4.55
BP	Stress response to metal ion	2	2.53	2.33E-02	83.71
BP	Detoxification of inorganic compound	2	2.53	2.33E-02	83.71
BP	Regulation of hydrolase activity	9	11.39	2.38E-02	2.52
BP	Cell communication	34	43.04	2.39E-02	1.37
BP	Positive regulation of calcium ion import	3	3.80	2.54E-02	11.96
BP	Regulation of extrinsic apoptotic signaling pathway	4	5.06	2.55E-02	6.24
BP	Cation transport	9	11.39	2.58E-02	2.48
BP	Regulation of ion transport	7	8.86	2.66E-02	3.02
BP	Cellular response to stimulus	38	48.10	2.68E-02	1.32
BP	Negative regulation of cellular process	26	32.91	2.71E-02	1.48
BP	Viral process	7	8.86	2.80E-02	2.98

BP	Cell activation	9	11.39	2.83E-02	2.44
BP	Cellular response to lipid	7	8.86	2.86E-02	2.97
BP	Multi-organism cellular process	7	8.86	2.90E-02	2.96
BP	Regulation of protein metabolic process	17	21.52	2.92E-02	1.72
BP	Positive regulation of cell migration	6	7.59	3.08E-02	3.38
BP	Signal transduction in response to DNA damage	3	3.80	3.17E-02	10.61
BP	Homeostatic process	13	16.46	3.30E-02	1.91
BP	Regulation of protein transport	8	10.13	3.38E-02	2.56
BP	Regulation of localization	17	21.52	3.40E-02	1.69
BP	Humoral immune response	4	5.06	3.43E-02	5.55
BP	Positive regulation of cell motility	6	7.59	3.48E-02	3.27
BP	Regulation of apoptotic cell clearance	2	2.53	3.48E-02	55.81
BP	Regulation of vasoconstriction	3	3.80	3.51E-02	10.05
BP	Negative regulation of biological process	27	34.18	3.58E-02	1.43
BP	Oxoacid metabolic process	8	10.13	3.65E-02	2.52
BP	Cellular response to drug	3	3.80	3.68E-02	9.78
BP	Positive regulation of cellular metabolic process	18	22.78	3.71E-02	1.63
BP	Cellular response to interferon-gamma	3	3.80	3.77E-02	9.66
BP	Positive regulation of cellular component movement	6	7.59	3.79E-02	3.19
BP	Positive regulation of locomotion	6	7.59	3.85E-02	3.18
BP	Positive regulation of heterotypic cell-cell adhesion	2	2.53	3.86E-02	50.23
BP	Blood vessel development	7	8.86	3.98E-02	2.74
BP	Positive regulation of macromolecule metabolic process	18	22.78	4.00E-02	1.62
BP	Regeneration	4	5.06	4.14E-02	5.15
BP	Signal transduction	31	39.24	4.20E-02	1.35
BP	Reproductive structure development	6	7.59	4.34E-02	3.08
BP	Regulation of chemotaxis	4	5.06	4.35E-02	5.05
BP	Negative regulation of angiogenesis	3	3.80	4.40E-02	8.86
BP	Reproductive system development	6	7.59	4.49E-02	3.04
BP	Cellular transition metal ion homeostasis	3	3.80	4.59E-02	8.66

BP	Cellular response to zinc ion	2	2.53	4.61E-02	41.86
BP	Establishment of blood-brain barrier	2	2.53	4.61E-02	41.86
BP	Regulation of establishment of protein localization	8	10.13	4.63E-02	2.39
BP	Negative regulation of blood vessel morphogenesis	3	3.80	4.69E-02	8.56
BP	Positive regulation of GTPase activity	4	5.06	4.79E-02	4.85
BP	Hormone secretion	5	6.33	4.79E-02	3.61
BP	Lipid metabolic process	10	12.66	4.80E-02	2.06
BP	Regulation of body fluid levels	5	6.33	4.84E-02	3.60
BP	Vasoconstriction	3	3.80	4.88E-02	8.37
BP	Phosphate-containing compound metabolic process	18	22.78	4.96E-02	1.58
BP	Vasculature development	7	8.86	4.96E-02	2.60
BP	Cell aging	3	3.80	4.98E-02	8.28
CC	Extracellular region	27	34.18	2.88E-10	4.09
CC	Extracellular space	25	31.65	4.09E-10	4.42
CC	Extracellular exosome	25	31.65	2.02E-05	2.48
CC	Integral component of plasma membrane	10	12.66	2.31E-02	2.36
MF	Chemokine activity	5	6.33	4.05E-05	25.60
MF	Chemokine receptor binding	5	6.33	9.78E-05	20.48
MF	Cytokine activity	7	8.86	3.19E-04	7.48
MF	Receptor binding	17	21.52	3.71E-04	2.67
MF	G-protein coupled receptor binding	7	8.86	7.44E-04	6.37
MF	Cytokine receptor binding	7	8.86	1.18E-03	5.83
MF	Secondary active transmembrane transporter activity	6	7.59	2.92E-03	6.07
MF	Active transmembrane transporter activity	7	8.86	4.17E-03	4.53
MF	Drug transporter activity	3	3.80	5.22E-03	27.30
MF	Enzyme inhibitor activity	7	8.86	5.40E-03	4.29
MF	Glycosaminoglycan binding	5	6.33	7.95E-03	6.27
MF	Peptidase inhibitor activity	5	6.33	1.13E-02	5.66
MF	CCR chemokine receptor binding	3	3.80	1.18E-02	17.98
MF	Transporter activity	12	15.19	1.19E-02	2.32

MF	Peptidase regulator activity	5	6.33	1.85E-02	4.88
MF	Symporter activity	4	5.06	2.02E-02	6.83
MF	Oxidoreductase activity, acting on paired donors, with incorporation or reduction of molecular oxygen, reduced flavin or flavoprotein as one donor, and incorporation of one atom of oxygen	3	3.80	2.13E-02	13.16
MF	Heparin binding	4	5.06	2.28E-02	6.51
MF	Substrate-specific transporter activity	10	12.66	2.67E-02	2.30
MF	Organic anion transmembrane transporter activity	3	3.80	4.29E-02	8.99
MF	Transmembrane transporter activity	9	11.39	4.36E-02	2.24
MF	Anion transmembrane transporter activity	4	5.06	4.36E-02	5.04
MF	Endopeptidase inhibitor activity	4	5.06	4.82E-02	4.84
KEGG	Chemokine signaling pathway	5	6.33	9.27E-03	5.79
KEGG	TNF signaling pathway	4	5.06	1.10E-02	8.33
KEGG	Complement and coagulation cascades	3	3.80	4.15E-02	8.96
